# Pulsed field ablation for the interventional treatment of atrial fibrillation: a scientific statement of the European Heart Rhythm Association of the European Society of Cardiology, the Heart Rhythm Society, the Asia Pacific Heart Rhythm Society, the Latin American Heart Rhythm Society, and the Canadian Heart Rhythm Society

**DOI:** 10.1093/europace/euag080

**Published:** 2026-04-12

**Authors:** Michael Kühne, Patrick Badertscher, Jason G Andrade, Ante Anic, Julian Chun, Antonio Dello Russo, Pierre Jais, Josef Kautzner, Heiko Lehrmann, Claire Martin, Jose Luis Merino, Damijan Miklavcic, Andrea Natale, Kars Neven, Armando Perez-Silva, Jonathan Piccini, Tobias Reichlin, Raphael Rosso, Martin Ruwald, Andrea Sarkozy, Daniel Scherr, Arian Sultan, Hiroshi Tada, Stylianos Tzeis, Atul Verma, Bradley Wilsmore, Helmut Pürerfellner

**Affiliations:** Department of Cardiology, University Hospital Basel, Petersgraben 4, Basel 4031, Switzerland; Department of Cardiology, University Hospital Basel, Petersgraben 4, Basel 4031, Switzerland; Department of Medicine, Vancouver General Hospital, Vancouver, Canada; Department for Cardiovascular Diseases, University Hospital Centre Split, Split, Croatia; Department of Cardiology, Cardioangiologisches Centrum Bethanien (CCB), Frankfurt am Main, Germany; Cardiology and Arrhythmology Clinic, Marche University Hospital, Ancona, Italy; Hôpital Cardiologique du Haut-Lévèque, CHU Bordeaux, LIRYC, Université de Bordeaux, Bordeaux, France; Department of Cardiology, Institute for Clinical and Experimental Medicine (IKEM), Prague, Czechia; Department of Cardiology, Faculty of Medicine, University Heart Center Freiburg-Bad Krozingen, University Hospital Freiburg, Freiburg im Breisgau, Germany; Cardiology Department, Royal Papworth Hospital, Cambridge, UK; La Paz University Hospital Research Institute (IdiPAZ), Universidad Autónoma de Madrid, Madrid, Spain; Faculty of Electrical Engineering, University of Ljubljana, Ljubljana, Slovenia; Texas Cardiac Arrhythmia Institute, St David’s Medical Center, Austin, USA; Department of Electrophysiology, Alfried Krupp Hospital, Essen, Germany; Department of Cardiology, Hospital Regional de Concepcion, Concepcion, Chile; Department of Medicine, Division of Cardiology, Duke Clinical Research Institute, Durham, USA; Department of Cardiology, Inselspital Bern, University of Bern, Bern, Switzerland; Tel Aviv Sourasky Medical Center, Tel Aviv University, School of Medicine, Tel Aviv, Israel; Department of Cardiology, Copenhagen Heart Center, Copenhagen, Denmark; Heart Rhythm Management Centre, Universitair Ziekenhuis Brussel, Heart Rhythm Research Brussels, Postgraduate Program in Cardiac Electrophysiology and Pacing, Vrije Universiteit Brussel, European Reference Networks Guard-Heart, Brussels, Belgium; Division of Cardiology, Department of Internal Medicine, Medical University of Graz, Graz, Austria; St.Georg Heart Center Hamburg, Asklepios Clinic Hamburg, Hamburg, Germany; Department of Cardiovascular Medicine, Faculty of Medical Sciences, University of Fukui, Fukui, Japan; Department of Cardiology, Mitera Hospital, Athens, Greece; Department of Medicine, McGill University Health Center, Montreal, Canada; Private Practice, Merewether, New South Wales, Australia; Department of Cardiology, Ordensklinikum Linz Elisabethinen, Linz, Austria

**Keywords:** Pulsed field ablation, Atrial fibrillation, Electroporation

## Abstract

Pulsed field ablation has emerged as a novel non-thermal treatment modality with a distinct safety profile for the interventional treatment of atrial fibrillation. By inducing irreversible electroporation, pulsed field ablation achieves myocardial ablation while preserving surrounding structures such as nerves, vasculature, and the oesophagus. This European Heart Rhythm Association of the European Society of Cardiology scientific statement, endorsed by major international societies, reviews the biophysics, technology, clinical evidence, workflow, safety, and training aspects of pulsed field ablation. Randomized trials demonstrate comparable efficacy to radiofrequency and cryoballoon ablation, with advantages in safety and efficiency. The statement provides practical advice for clinical implementation and operator training and identifies key gaps in evidence and priorities for future research and innovation.

## Table of contents

Introduction
Biophysics of pulsed field ablation
 Background and development
 Mechanism of action
 Comparison with other ablation technologies
 Biomarker release
Technological considerations
 Available pulsed field ablation platforms
 Energy delivery and dosing
 Dual-energy (pulsed field ablation/radiofrequency ablation) ablation systems
Patient selection and procedural considerations
 Primary uses of pulsed field ablation
  Clinical context of de novo atrial fibrillation ablation
  Repeat atrial fibrillation ablation procedures
 Clinical considerations limiting pulsed field ablation use
Procedural workflow
 Pre-procedural preparation
  Role of general anaesthesia and sedation protocols
  Role of imaging
  Fluoroscopy-only standardized workflow
  Role of 3D electroanatomical mapping
  Pulsed field ablation in patients with cardiac implantable electronic devices
  Pulsed field ablation in patients with left atrial appendage occlusion
  Concomitant pulsed field ablation and left atrial appendage occlusion
  Pulsed field ablation in patients with other intracardiac devices
 Step-by-step procedure
  Vascular access
  Choice of guidewire
  Transseptal puncture
  Sheath/catheter exchanges and air embolism prevention
  Vagal response during ablation
  Assessment of catheter-tissue contact
  Pulsed field ablation dosing strategies
  Angiographic projections
  Role of intracardiac echocardiography/zero-fluoroscopy workflow
  Troubleshooting (energy interruption, electrical arcing)
 Procedural endpoint definition
 Post-procedural care/same-day discharge protocols
Efficacy and safety
 Clinical outcomes
  Randomized controlled trials
  Single-arm registries
  Blanking period
Occurrence and management of adverse events
 Pulsed field ablation-related adverse events
  Haemolysis/acute kidney injury
  Phrenic nerve injury
  Coronary injury/drug protocols for prevention of vasospasm
  Pulmonary vein stenosis
  Oesophageal events
  Conduction system disturbances
  Pericarditis
  Effect on left atrial function
 Non-pulsed field ablation-related events
  Pericardial tamponade
  Stroke/transient ischaemic attack/cerebral emboli
  Vascular access complications
  Death
 Autonomic nervous system effects
 Long-term follow-up data
Training and education
 Structured training pathway
 Learning curve and competency milestones
 Integration into electrophysiology fellowship programmes
 Non-technical skills and professional standards
Future directions
 Innovations in pulsed field ablation technology
  Focal vs large footprint innovations and dual-energy ablation
  3D mapping integration, real-time signal analysis, lesion- effect markers
 Ongoing research and clinical trials
 Emerging applications for pulsed field ablation
  Posterior wall isolation
  Superior vena cava isolation
  Mitral isthmus line
  Left atrial appendage isolation
  Scar homogenization
  Use in typical atrial flutter
  Use in ventricular arrhythmias
  Use in supraventricular tachycardia
 Gaps in evidence
Writing committee position
 Summary of key points
 Conclusion
Acknowledgements
Data availability
References

## Introduction

Catheter ablation has become a cornerstone in the treatment of atrial fibrillation (AF), aiming to achieve durable pulmonary vein isolation (PVI) and thereby eliminate arrhythmogenic triggers within the pulmonary veins (PVs). For over two decades, radiofrequency (RF) and then cryothermal ablation have been the predominant energy sources used for AF ablation. While effective, these thermal modalities rely on tissue heating or freezing, potentially resulting in collateral injury to surrounding structures such as the oesophagus and the phrenic nerve. Despite continuous refinements in technology and workflow, major complications such as atrio-oesophageal fistula (AEF) and PV stenosis remain rare but potentially fatal, whereas phrenic nerve palsy, although generally non-fatal, is associated with significant morbidity. These limitations have driven the search for alternative, safer, and more selective energy sources.

Pulsed field ablation (PFA) has emerged as a novel ablation modality based on the principle of irreversible electroporation. Pulsed field ablation disrupts cell membrane integrity through the delivery of high-voltage, short-duration electrical pulses. These pulses create nanoscale pores, alter transmembrane potential, and disturb cellular homeostasis, ultimately leading to cell death. Because myocardial tissue has a lower electroporation threshold than surrounding structures, PFA may allow relatively selective myocardial injury.^1^ The myocardial sensitivity and short energy application duration of PFA translate into an improved safety profile and the potential to simplify ablation workflows. In preclinical and clinical studies, PFA has demonstrated durable lesion formation, minimal thermal effect, and preservation of extracardiac structures.

Since its first clinical applications, PFA has progressed from early feasibility to widespread adoption. Multiple catheter platforms and generators are now available, including multi-spline catheters, circular catheters, large-footprint lattice-tip catheters, balloon systems, focal solid-tip catheters, and dual-energy designs that combine both PFA and RF delivery. Randomized controlled trials and large registries have confirmed the clinical efficacy of PFA to be at least equivalent to that of thermal ablation, while procedure times are generally shorter and complication rates—particularly oesophageal and phrenic nerve injury (PNI)—are lower. Whether PFA fundamentally alters ablation strategies beyond PVI, improves durability at non-PV targets, or safely expands the range of atrial substrate that can be treated by ablation remains an area of active investigation.^2^

Given the rapid clinical uptake of PFA, comprehensive practical guidance on its principles, procedural application, safety considerations, and associated training requirements is needed. This scientific statement by the European Heart Rhythm Association (EHRA) of the European Society of Cardiology (ESC) and collaborating partner societies worldwide summarizes current evidence, provides practical guidance for clinical use and training, and outlines priorities for future research to ensure safe and standardized global implementation of PFA in the interventional treatment of AF.

## Biophysics of pulsed field ablation

### Background and development

**Figure 1:**
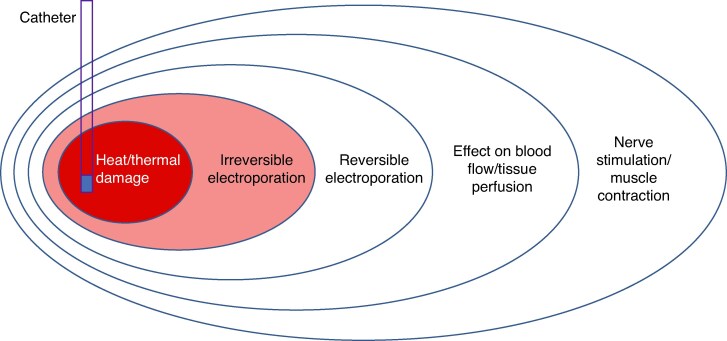
Each waveform produces distinct tissue responses in the region surrounding the catheter. In the immediate vicinity, the highest electric field strength and current density result in thermal injury, specifically coagulative necrosis. This is followed by zones of irreversible electroporation, then reversible electroporation at greater distances, which leads to cellular stunning and transient effects on vasculature and perfusion. At the greatest distances from the catheter, nerve stimulation is expected, involving both autonomic nerves and neuromuscular capture.

The year 2007 marked an important milestone in the development of PFA, which gained significant interest due to two independent publications.^3,4^ The first PFA systems were using DC/monophasic pulses, either exponentially decaying or monophasic 100 µs pulses (*Figure 1*). The first PFA systems were developed for PVI, creating lesion depths of 2–5 mm. Currently, most of the systems can achieve these depths easily but when tested in ventricles with thicker tissue they can only achieve lesion depths of 6–8 mm, irrespective of different waveforms and catheter designs.^5^ In general, the waveform parameters remain mostly undisclosed as proprietary information, which makes comparison of PFA systems and collective learning difficult.

### Mechanism of action

**Figure 2:**
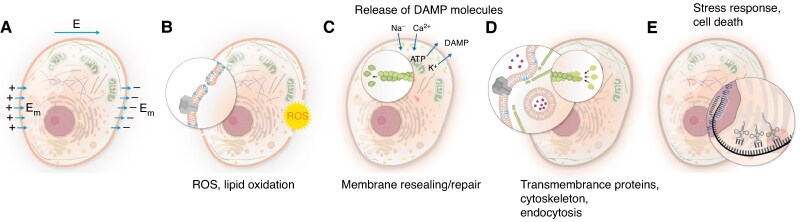
Electroporation can be achieved using a variety of pulse shapes and parameter settings: duration (ns, µs, ms), number of pulses or trains, and shape (e.g. mono-, biphasic, exponentially decaying, sinusoidal). Despite these differences, all electroporation modalities share common hallmarks: induced transmembrane voltage (comparable to that responsible for action potentials) leading to transient transmembrane protein damage and lipid oxidation. This process permits the transport of ions and molecules across the membrane, including calcium influx (causing calcium overload and disruption of the cytoskeleton), sodium influx, and potassium efflux. The resulting membrane depolarization produces cellular non-excitability and stunning, while the release of damage-associated molecular patterns (DAMPs) triggers an immune response. The outcome ultimately depends on membrane repair, leading either to cell recovery or to cell death. ROS, reactive oxygen species.

When high-voltage pulses are delivered between two or more electrodes, an electric field and current (density) are established in the tissue. The electric field is the highest close to the electrode and decreases rapidly with distance. Similarly, current density is the highest around the electrodes, in particular at sharp edges and between neighbouring electrodes on the catheter. Current flow through the tissue causes tissue heating, albeit significantly less in magnitude and duration than the resistive heating associated with RF energy. At low frequencies, current mainly flows through the extracellular milieu. The lipid bilayer of the cell membrane is poorly conductive and acts as a barrier to electrical current. As a result, a potential difference is built across the membrane (e.g. 0.5 V), amplifying the electric field. This leads to a high field within the membrane (0.5 V over 5 nm distance, 10^8^ MV/m). This field causes pore formation, allowing water and reactive oxidative species (ROS) to reach into the hydrophobic domain of the lipid bilayer. This causes lipid oxidation and damages transmembrane proteins—all together contributing to sustained increased membrane permeability.^6^ Increased membrane permeability for molecules otherwise deprived of transmembrane transport mechanisms also results in often only transient increased conductivity to ions (e.g. Na, K, Ca) and can affect a significantly larger area, rendering cells in this area unexcitable and unable to propagate action potentials. This phenomenon is also reflected by a rapid and significant decrease of bipolar EGM signals with PFA.^7^ Membrane resealing and cell recovery, however, render some of these cells excitable within minutes.^8^ Repair of the membrane and recovery of cells may require up to several minutes, depending on the extent of electroporation and damage inflicted to the cell membrane but can be quite unpredictable.^9^ Since sufficiently high electric fields can electroporate any cell, this may result in stunning and damage of nerves and conduction system.^10–14^ If the cell does not recover, cell death due to loss of homeostasis occurs (irreversible electroporation) which does not necessarily occur instantaneously. Cell death pathway and its dynamics may depend on the waveform and amplitude (*Figure 2*).^15^

In addition to membrane effects, electroporation induces transient microvascular changes, including temporary capillary flow reduction and increased endothelial permeability.^16–18^ These effects represent functional and largely reversible alterations in vascular tone and endothelial integrity rather than structural damage to the extracellular matrix (ECM), which remains preserved. Increased permeability may lead to transient oedema formation.^19^ The resulting transient disruption of oxygen and nutrient supply to the tissue may further contribute to lesion formation. Since electroporation is a membrane phenomenon, it does not affect ECM, provided there is no significant increase in temperature. Factors such as the availability of blood supply (preservation of microcirculation) and the ECM facilitate tissue repair. Notably, lesion development and maturation in PFA appear to occur more quickly compared to RF and cryoablation (*Table 1*).^20^

**Table 1:** Comparison of various ablation energies and their effects on cells and tissue

Ablation method	Energy delivery and propagation	Cell damage	(Micro) vasculature	Extracellular matrix	Healing
Radiofrequency ablation (thermal/heating)	Volumetric heating and thermal diffusion	Indiscriminate denaturation of proteins	Highly thrombogenic, indiscriminate thermal damage	Coagulation	Delayed, from periphery
Cryoablation (freeze-thaw)	Thermal diffusion	Crystal formation; cell membrane damage; osmotic imbalance	Clogging and damage of microvasculature	Preserved	Delayed, from periphery
Pulsed field ablation (electroporation)	Electric field effect and volumetric heating	Membrane lipid oxidation and protein damage	Preserved with transient reduction of blood perfusion and increase microvascular permeability	Preserved	Enabled/facilitated, lesion accessible by preserved microcirculation

During the delivery of high-voltage pulses (mostly ≤3000 V, 10–30 A), significant instantaneous power is delivered. In areas where the electric field and current density are highest, catheter and tissue can heat up. Depending on the duty cycle (i.e. how much energy is delivered per unit of time), significant temperature increases can be achieved with varying degrees of thermal damage to the tissue.^19,21^ The damage of tissue in the immediate vicinity of the catheter is therefore likely to be thermal in nature indicating that during PFA the endothelium may be damaged thermally,^22^ as may the phrenic nerve in case of epicardial ablation.^21^ Lesion shape and size can differ, depending on the mode of PFA delivery (monopolar vs. bipolar) and characteristics of the catheter (solid tip, large focal vs. multipolar).^23^ The characteristics of the delivery catheter also affect the required contact with the tissue, enabling the formation of contiguous lesions even in trabeculated areas.^24–26^ Furthermore, as blood is a better conductor than tissue, the electric field extends into the blood pool causing electroporation of blood cells. With an increasing number of applications, significant haemolysis has been observed (see Section Haemolysis/acute kidney injury ). Finally, electroporation has been demonstrated *in vitro* to induce platelet activation and modulate neutrophil activation.^27,28^

### Comparison with other ablation technologies

Pulsed field ablation destroys cells within the tissue by overwhelming their capacity to recover from membrane damage and disruption of cell homeostasis (*Table 1*). Electroporation transiently increases membrane permeability, allowing molecules and ions to cross the plasma membrane, which consequently leads to cell death. Although the exact dynamics and mechanisms of cell death are not entirely clear, a massive influx of Ca and ATP depletion are contributing factors or are at least hallmarks of cell death. In contrast, RF creates irreversible tissue injury when resistive or conductive heating results in tissue temperatures greater than 50°C leading to protein denaturation and coagulative necrosis, while cryoenergy leads to irreversible cell death via freeze-thaw cycling, disrupting cell membranes and inducing ischaemia.

### Biomarker release

Due to the different mechanism of action of PFA, but also probably because of a greater extent of ablation, biomarkers exhibit different temporal dynamics and levels compared to RF and cryoablation. Recent studies have shown significant differences in the dynamics and elevated levels of biomarkers indicative of myocardial injury (i.e. hs-cTnT, myoglobin, CK-MB) after PFA compared to RF ablation.^29^ Furthermore, differences seem to exist between different PFA systems, which may in part relate to variations in catheter design, including electrode surface area, geometry, and energy delivery characteristics that influence blood–electrode interaction and field distribution. Importantly, reduced glomerular filtration can amplify these effects.^30^ A recent systematic review of available studies suggested that the severity of haemolysis correlates with procedural factors and catheter design.^31^ The increase of inflammation biomarkers (i.e. CRP), following PFA and RF, was found to be similar. However, it was observed that CRP normalization occurred more rapidly after PFA than after RF. Markers of renal function and electrolyte balance exhibited only minor fluctuations over time, remaining within the expected physiological range for PFA and RF.^32^

## Technological considerations

### Available pulsed field ablation platforms

Contemporary PFA catheter technology can be categorized into three main groups. Circumferential PVI tools are large-area or balloon-based systems designed for rapid and efficient PVI (*Table 2*). Their strengths lie in procedural simplicity, short ablation times, and robust safety data.

**Table 2:** Representative PFA catheter platforms. Devices are categorized by catheter architecture and ablation strategy. Multielectrode ‘single-shot’ systems are primarily designed for pulmonary vein isolation, whereas focal catheters allow point-by-point ablation similar to conventional RF technology. Differences in energy delivery (monopolar vs. bipolar) and waveform characteristics contribute to variability between platforms

	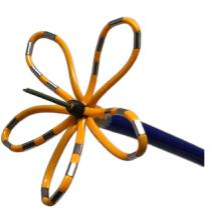	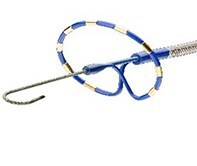	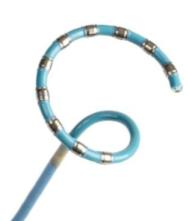
	Farapulse™	PulseSelect™	Varipulse™
	Boston Sci	Medtronic	Biosense Webster
Diameter	31/35 mm	25 mm	25–35 mm
Size	12 F	9 F shaft/9.8 F at array/shaft bond	8.5 F
Over the wire	Yes	Yes	No
Pulse amplitude/duration	1.8–2.0 kV/µs	1.5 kV/µs	1.8 kV/µs
Ablation mode	Bipolar	Bipolar	Bipolar
Wave description	Biphasic	Biphasic	Biphasic
PFA impulse duration	Micro- to sub-millisecond	Microseconds	Microseconds
PFA application duration	2.5 s for a single application 8 minimum per PV advised	4–5 s per application 8 minimum per PV advised	∼22 s per ablation (1 ablation = 3 applications) 4 ablation per PV advised
Contact sensing/type	No	No	No
Dedicated 3D mapping	Yes	Yes	Yes
Approval	EU/USA/Japan/Australia	EU/USA/Japan/Australia	US/EU/APAC/LATAM

	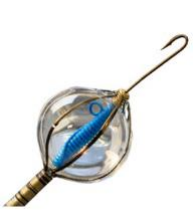	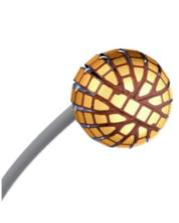	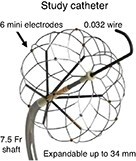
	Volt™	Globe PF™	Sphere 360™
	Abbott	Kardium	Medtronic
Diameter	28 mm	30 mm	34 mm
Size	13 F	16 F	8 F
Over the wire	Yes	No	Yes
Pulse amplitude/duration	1.5–1.8 kV/µs	1.7 kV/µs	1.3–2.0 kV/µs
Ablation mode	Bipolar	Bipolar	Monopolar
Wave description	Biphasic	Biphasic	Biphasic
PFA impulse duration	Microseconds	Microseconds	Microseconds
PFA application duration	∼20 s (10 bursts R wave gated)	2 s per train Single application per vein/target area 2–6 trains per application	5–6 s per application 4 minimum per PV advised
Contact sensing/type	Yes/impedance	Yes/thermal	No
Dedicated 3D mapping	Yes	Yes	Yes
Approval	EU/USA	USA	EU

	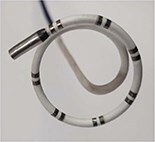	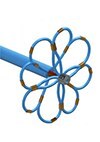	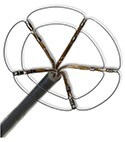	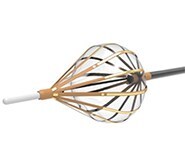	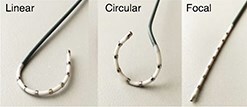
	Adagio™	Lotos™	nsPFA™	Optishot™	SineBurst™
	Adagio	Lifetech	Pulse Bioscience	CardioFocus	Arga Medtech
Diameter	25 mm variable	28/31 mm	30 mm	Up to 40 mm	25 mm
Size	8.5 F	12 F	11 F	12 F	7 F
Over the wire	No	No	No	No	No
Pulse amplitude/duration	1.1 kV/µs	1.85–2.1 kV/µs	? kV/ns	2.0 kV/µs	2.25–3.25 kV/µs
Ablation mode	Bipolar	Bipolar	Bipolar	Bipolar	Bipolar, monopolar, combination
Wave description	Biphasic	Biphasic	Monophasic	Biphasic	Biphasic, coherent sine-wave
PFA impulse duration	Micro- to sub-millisecond	Nanosecond	Nanosecond	Microseconds	Microseconds
PFA application duration	Single set of 15 pulse trains between sequential electrodes, preceded by 30 s ultra-low temperature ablation	5–11 s, 5 trains per site	5 s	25 s (1–2 app/PV)	13 s for nominal dose delivered through all 8 electrodes; phase shifting or maximum output prolongs application
Contact sensing/type	No	No	No		
Dedicated 3D mapping	No	No	No	No (not required)	No
Approval	No	No	No	No	No

The second group comprises large-footprint catheters that integrate with 3D electroanatomical mapping (EAM) systems (*Table 3*). These devices can be used both for PVI and linear ablation, and potentially individualized lesion sets guided by 3D mapping. Their principal advantage is adaptability and the ability to create broad lesion sets in both atrial and ventricular tissue, while limitations include the need for advanced mapping, limited clinical experience to date, and the ongoing development of reliable contact and lesion assessment tools.

**Table 3:** Representative of large-footprint PFA catheters integrated with 3D electroanatomical mapping systems

	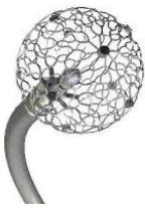	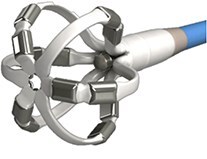	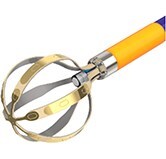	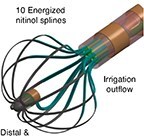
	Sphere-9™	Omnypulse™	Faraflex™	QuickShot™
	Medtronic	Biosense Webster	Boston Scientific	CardioFocus
Diameter	9 mm	12 mm	9 mm	10 mm
Size	8 F	7.5 F	8 F	8.5 F
Steerability	Bidirectional	Bidirectional	Bidirectional	Bidirectional
Pulse amplitude/duration	2 kV/µs (Pfrev: 32 A/<10 µs, EU only)	1.2–1.5 kV/µs	2 kV/µs	3.5 kV/µs
PFA mode	Monopolar	Bipolar	Monopolar/bipolar	Monopolar
Wave description	Biphasic	Biphasic	Biphasic	Biphasic
PFA impulse duration	Microseconds	Microseconds	Microseconds	Microseconds
PFA application duration	4 s	Up to 12 applications max (14.5 s for 12 apps), but duration varies according to PF index target value	2.5 s	QS: 3 s QS+: 6.8 s
RFC/PFA	Yes/yes	No/yes	No/yes	No/yes
Dedicated 3D mapping	Yes	Yes	Yes	Open platform
Approval	EU/USA	No	No	No

The third group includes systems that use conventional small-tip RF catheters to deliver PFA through established contact force mapping and ablation catheters (*Table 4*). This approach combines the flexibility of point-by-point lesion creation with the familiarity of standard workflows and is suitable for both atrial and ventricular arrhythmia ablation. Advantages include versatility, individualized lesion sets, and integration into existing electrophysiology labs. Limitations are that these systems require ‘point-by-point’ operator skills, which may translate into longer or workflow-dependent procedures for PVI, while PFA energy delivery parameters still require optimization for some systems that are in early clinical evaluation. Of note, multiple novel devices are currently under development; therefore, no complete list can be obtained. Details of current catheter technologies are summarized in *Tables 2–4*. Given the substantial heterogeneity in PFA catheter design and terminology, harmonization is needed to facilitate comparison across studies and platforms; current PFA catheters can be grouped accordingly, and a recent consensus proposal suggests classifying systems based on the largest dimension in contact with tissue as regional (>12 mm), large-tip focal (>4–≤12 mm), or focal (≤4 mm; *Table 5*).

**Table 4:** Representative of PFA systems based on conventional small-tip RF catheters integrated with contact force sensing and electroanatomical mapping

	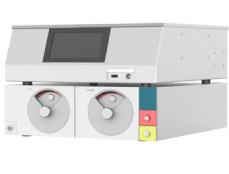	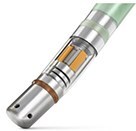	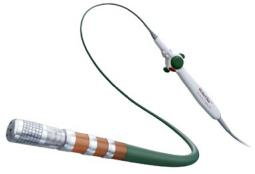	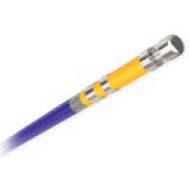
	Centauri™	STSF Dual Energy™	Tactiflex Dual Energy™	Farapoint™
	CardioFocus	Biosense Webster	Abbott	Boston Scientific
Electrode spacing	3.5–4 mm	3.5 mm	4 mm	3.5–4 mm
Size	8 F	8 F	8 F	8 F
Steerability	Uni-/bidirectional	Bidirectional	Bidirectional	Bidirectional
Contact force sensing	Yes	Yes	Yes	No, local impedance (future)
Amplitude/duration	19–25 A/µs	(Pulse amplitude) 1.0–1.5 kV/µs	1.9–2.2 kV/µs	1.4–2.0 kV/µs
RFC/PFA	No/yes	Yes/yes	Yes/yes	No/yes
PFA impulse duration	Microseconds	Microseconds	Microseconds	Microseconds
PFA application duration	19 A: 3 s 22 A: 5 s 25 A: 7 s	Up to 24 applications (max 28s for 24 apps), but duration varies according to PF index target value	2.2 kv: ∼5 s 1.9 kV: ∼10 s	2.5 s
Ablation mode	monopolar	Monopolar	Monopolar	Bipolar
Wave description	biphasic	Biphasic	Biphasic	Biphasic
Dedicated 3D mapping	Open platform	Yes	Yes	Yes
Approval	EU	EU	EU, Dec 2025	EU (and US approval CTI indication)

**Table 5:** Suggested framework for harmonized classification of PFA catheters

Catheter classification	Proposed definition	Previous terms used
Regional catheter	Catheter with the largest dimension in contact with tissue > 12 mm	Circumferential PVI catheter; single-shot catheter
Large-tip focal catheter	Catheter with the largest dimension in contact with tissue > 4 mm and ≤ 12 mm	Wide-area focal catheter; large-tip or lattice-tip catheter (e.g. Affera); large-tip focal catheter (e.g. Omnipulse)
Focal catheter	Catheter with the largest dimension in contact with tissue ≤ 4 mm	Conventional focal catheter; large/intermediate footprint catheter; focal catheter

### Energy delivery and dosing

Energy delivery in PFA is defined by pulse amplitude (typically 500–3000 V), pulse duration (nanosecond to microsecond range), pulse number, repetition frequency, and waveform (monophasic vs. biphasic, monopolar vs. bipolar, square vs. sine wave). These parameters determine the biological effect at the cellular level and drive lesion size and shape. Bipolar delivery and biphasic waveforms can reduce charge build-up and muscle stimulation, while monopolar delivery and monophasic waveforms typically produce deeper lesions. Catheter design—including electrode geometry, interelectrode spacing, and multielectrode arrays—shapes and affects the electric field distribution, lesion morphology, and its consistency. Irrigated vs. non-irrigated designs influence thermal management rather than primary lesion mechanism. Synchronized delivery with the cardiac cycle (ECG gating) is abandoned in most of the platforms currently in clinical use for atrial ablations due to low arrhythmogenicity of biphasic pulses as demonstrated in large clinical studies.^33^

Delivery strategies vary: focal or large-focal point-by-point PFA with steerable catheters for linear or focal lesions, and multielectrode systems with diverse geometries for rapid PVI. Safety considerations include preventing unintended arcing, monitoring for conduction system capture, avoiding excessive overlap of high-intensity fields near critical structures, and adherence to device-specific dosing protocols derived from preclinical and clinical data.

### Dual-energy (pulsed field ablation/radiofrequency ablation) ablation systems

Dual-energy ablation systems combine RF and PF technologies within a single device. Radiofrequency ablation (RFA) and PFA delivery is integrated through unified power management and control architecture. The electrode array of the catheter is connected to a generator capable of switching between RF output and biphasic or monophasic high-voltage PF trains. Internal solid-state switching circuits or H-bridge inverters reconfigure the output stage to match the impedance and waveform requirements of each mode. During operation, impedance, temperature, and voltage sensors embedded in the catheter provide continuous feedback to the generator's microcontroller. This feedback loop governs energy delivery, preventing excessive tissue heating during RF mode, and provides over-current protection and reliable pulse delivery.

One dual-energy system (Affera Sphere-9, Medtronic) comprises a 9 mm tip sphere catheter, formed by an expandable nitinol lattice.^34^ Another system (ThermoCool SmartTouch SF Dual Energy Catheter and TRUPULSE Generator, Johnson & Johnson) uses an existing contact force RF catheter (SmartTouch SF) modified to deliver both RFA and PFA energy using a single catheter/generator combination and integrates with the CARTO-3 mapping system. Other systems are under investigation.

Dual-energy ablation platforms are designed to harness the complementary advantages of PFA and RFA to maximize procedural safety, versatility, and efficacy. A dual-energy system allows operators to tailor energy delivery based on anatomy, where PFA may be used for thin-walled structures, while RFA is suitable for thicker myocardium and in locations where coronary artery spasm may be a concern. A combination of PF and RF lesions may be employed to create a deeper lesion.^35,36^ The ability to switch between PFA and RFA modes within the same catheter and generator may reduce the need for multiple catheters and catheter exchanges and may streamline procedures. For some ablations, this flexibility might potentially reduce procedure times, increase first-pass success, and reduce complications, but comparative outcome data is awaited.

## Patient selection and procedural considerations

### Primary uses of pulsed field ablation

#### Clinical context of *de novo* atrial fibrillation ablation

Pulsed field ablation for *de novo* paroxysmal AF has demonstrated clinical advantages, particularly in terms of safety, efficiency, and procedural consistency. To date, more than 500 000 procedures with the pentaspline PFA catheter have been performed worldwide without a single reported case of AEF.^37,38^ In addition, PV narrowing remains exceedingly rare with PFA and has not been linked to clinical symptoms to date (also see Section Efficacy and safety).^39,40^

Despite being a first-generation technology, PFA for ablation of paroxysmal AF already at least matches the efficacy of established thermal energy sources.^41,42^ In cases of early persistent AF with minimal atrial remodelling, PVI alone using PFA represents an appropriate strategy and can be employed using an approach analogous to that used for paroxysmal AF. In more advanced forms, a PVI-only strategy may still be acceptable but typically yields lower success rates, and additional lesion sets may be required. In persistent AF, evidence is emerging but remains less mature than in paroxysmal AF. The multicentre single-arm ADVANTAGE AF programme evaluated pentaspline PFA with PVI plus posterior wall (PW) ablation,^43^ and Phase 2 uniquely used continuous insertable cardiac monitor follow-up, reporting 1-year effectiveness rates of 65–75% and low adverse event rates.^44^ Similarly, the SPHERE Per-AF study demonstrated non-inferior efficacy and safety and increased efficiency of PFA over RF in patients with persistent AF.^43,45^ Based on the latest EHRA/HRS/APHRS/LAHRS Expert Consensus Document on Catheter and Surgical Ablation of Atrial Fibrillation,^46^ the role of additional lesion sets beyond PVI in patients with persistent AF remains an area of uncertainty. Ongoing randomized studies, such as PIFPAF-PFA (NCT05986526), are currently evaluating whether PW ablation using PFA improves outcomes in this population.

#### Repeat atrial fibrillation ablation procedures

Remapping series after index PFA ranged from very high PVI durability rates to comparable results as achieved by thermal ablation.^47–49^ In remapping studies, higher reconnection rates were observed at the LSPV and RIPV with potential differences between different PFA systems.^50–52^ Posterior wall isolation (PWI) using PFA has emerged as an adjunctive therapy, especially in the treatment of persistent AF. First data show high durability of PWI using PFA.^53^

The integration of 3D mapping into current PFA platforms has improved visualization of catheter position, tissue contact, and lesion sets, enabling a ‘mapping-on-the-fly’ workflow in which mapping occurs before, during, and after energy delivery. In redo procedures, however, the approach often differs, with many operators preferring an additional high-density mapping catheter to better delineate residual substrate. Redo ablation strategies may include repeat PVI alone, PWI, or adjunctive linear lesions such as a left atrial anterior line, mitral isthmus line, and/or cavotricuspid isthmus (CTI) line (see Section Coronary injury/drug protocols for prevention of vasospasm for risks and mitigation strategies).^54–56^ Despite some favourable data outcome data in redo procedures using PFA in the setting of persAF, larger prospective randomized data are still lacking, and the ideal ablation approach remains to be determined.^57,58^

### Clinical considerations limiting pulsed field ablation use

To our knowledge, no PFA-specific contraindications have been identified beyond the general contraindications to undergo catheter ablation. The use of PFA in specific subgroups, e.g. patients with cardiac implantable electronic devices (CIEDs) and patients with left atrial appendage occlusion (LAAO), is described in the following Sections: Pusled field ablation in patients with cardiac implantable electronic devices and Pulsed field ablation in patients with left atrial appendage occlusion.

## Procedural workflow

### Pre-procedural preparation

#### Role of general anaesthesia and sedation protocols

The choice between general anaesthesia (GA) and deep sedation has been a subject of discussion since the first-in-human electroporation studies. Early monophasic systems required GA because of strong skeletal muscle capture.^59,60^ With the introduction of biphasic waveforms, muscular contractions are significantly reduced, enabling most procedures to be performed under deep sedation.^61–63^ It should be noted that there remain differences between different systems in terms of muscular contractions.^62,64^ For clarity, GA is generally defined as anaesthesia requiring airway control and often neuromuscular blockade, whereas deep sedation refers to the administration of hypnotic and analgesic agents without neuromuscular blockade and typically without endotracheal intubation, while maintaining spontaneous ventilation. However, terminology across published studies has not always been used consistently. Large-scale registry data, such as the MANIFEST-PF survey^65^ (>17 000 patients), indicate that more than 80% of procedures are conducted with deep sedation alone, with favourable safety and efficacy outcomes.^52^ Sedation regimens typically combine propofol, benzodiazepines, and short-acting opioids, supporting streamlined early or same-day discharge workflows.^66,67^ An overview of common sedation protocols used for PFA is provided in *Table 6*. Administration of agents, such as propofol and dexmedetomidine, has traditionally required the presence of an anaesthesiologist, whereas in some jurisdictions, deep sedation (also using propofol and dexmedetomidine) can be administered by trained non-anaesthesiologists.

**Table 6:** Sedation and anaesthesia approaches for PFA (illustrative dosing)

Protocol	Drug(s)	Typical dosing	Notes
Local anaesthesia (adjunct only)	Local anaesthetic at access sites	Per institutional practice	Insufficient as sole strategy for PFA owing to discomfort and skeletal muscle contractions; used only as adjunct
Conscious (moderate) sedation^68^	Midazolam + short-acting opioid; multimodal adjuncts (e.g. lidocaine for coughing), optional bolus etomidate if required	Midazolam 0.01–0.03 mg/kg IV + fentanyl 0.5–1 µg/kg IV; lidocaine 1–1.5 mg/kg IV; optional etomidate bolus (e.g. 0.05–0.1 mg/kg)	Feasible for certain PFA systems in selected patients; without routine involvement of anaesthesiology personnel; preserves spontaneous respiration but may provide less immobility and comfort than deep sedation
Deep sedation^62^	Midazolam + short-acting opioid ± ketamine or dexmedetomidine	Midazolam 0.01–0.03 mg/kg IV; fentanyl 0.5–1.5 µg/kg IV; ketamine 0.5–1 mg/kg IV before first PFA, top-ups 0.1–0.2 mg/kg as needed	Increasingly preferred for PFA; preserves spontaneous respiration; high safety and short recovery when delivered by trained staff with structured monitoring
Propofol-based deep sedation^69^	Propofol + short-acting opioid ± small benzodiazepine	Propofol 0.5–1 mg/kg IV bolus, then 0.25–0.5 mg/kg boluses or 25–75 µg/kg/min infusion; fentanyl 0.5–1 µg/kg IV; optional midazolam 0.01–0.03 mg/kg IV at start or minimal-propofol strategy^66^: midazolam 0.01–0.03 mg/kg + fentanyl 0.5–1 µg/kg IV; propofol only in 0.25–0.5 mg/kg rescue boluses	Most commonly used in clinical routine; rapid onset; stable and predictable if titrated slowly; requires continuous monitoring and airway-trained personnel
General anaesthesia	Per anaesthesiology practice	Per institutional standards	Provides full airway control and immobility; suitable for complex or high-risk cases or patients with obesity/anticipated difficult airway; associated with longer lab occupancy and higher resource demand

Additional real-world evidence comes from the EU-PORIA registry, which enrolled 1233 consecutive patients across seven European centres. In this cohort, 20% of procedures were performed under GA and 80% under deep sedation. Deep sedation was associated with significantly shorter skin-to-skin and fluoroscopy times, and there were no differences in serious adverse events between groups. At 1-year follow-up, freedom from recurrent AF/AT was virtually identical (approximately 74%), supporting the clinical equivalence of both strategies when using the pentaspline PFA catheter.^70^

General anaesthesia may be appropriate in patients with poor tolerance of deep sedation or with airway management concerns due to obstructive sleep apnoea or obesity. It also facilitates immobility and catheter stability, particularly early in the learning curve. However, no randomized evidence demonstrated superiority of GA over sedation for PFA. Both approaches are used, and the choice should be individualized based on institutional practice, availability of GA, choice of PFA technology, operator preference, and patient comorbidity profile. Standardized monitoring, including continuous capnography, is advised according to local protocols when sedation is used.^71^ Of note, terminology regarding anaesthesia and sedation in prior studies has often been used inconsistently. Many procedures described as ‘deep sedation’ employ the same hypnotic and analgesic agents as GA, with the key distinction being the absence of neuromuscular blockade. Therefore, clearer reporting of anaesthetic strategies, including the sedative agents used and the application of neuromuscular blockade, would improve comparability across studies.

#### Role of imaging

Pre-procedural imaging of the left atrium with computed tomography (CT) or magnetic resonance imaging (MRI) is frequently obtained before PVI. Such imaging permits characterization of PV anatomy and may be fused with EAM or fluoroscopy to guide ablation.^72^ Since some catheters are available in more than one size, imaging may support device selection, although evidence for this approach is lacking.^73^ The impact of pre-procedural imaging on efficacy and safety in PFA remains uncertain. Computed tomography or MRI may be particularly useful in the early learning phase and in patients with complex anatomy. With growing operator experience, fluoroscopy-only workflows are safely used in high-volume centres. Of note, many centres still rely on intra-procedural pulmonary venous angiography for anatomical delineation.

Intracardiac echocardiography (ICE) offers real-time visualization of PV anatomy, rules out left atrial appendage (LAA) thrombus, guides safe transseptal puncture, confirms tissue contact of ablation catheters, and enables rapid detection of acute complications such as pericardial effusion.

#### Fluoroscopy-only standardized workflow

Fluoroscopy-only workflows are attractive because they simplify logistics, shorten pre-procedural planning, and reduce costs. The trade-off involves potentially increased dependence on radiation exposure, underscoring the importance of operator experience and adherence to radiation-sparing techniques. Recent evidence shows that fluoroscopy-only PFA shortens procedure times without affecting safety or outcomes.^74,75^ Catheter–tissue contact can be inferred by fluoroscopic spline indentation or change in circular shape. Catheter positioning may be further optimized by pacing from the PFA catheter prior to the first application and adjusting contact according to atrial capture.^76^

#### Role of 3D electroanatomical mapping

In the early development of PFA systems, no mapping capacities were available, and a multipolar catheter had to be used for pre- and post-procedural mapping. This approach required sheath exchange, increased procedural complexity, and costs, while the PFA catheter itself could not be visualized. Later, PFA catheters became displayable within 3D mapping systems via impedance-based localization, but only as simplified representations, limiting positioning accuracy. Today, PFA is increasingly integrated into EAM systems. An overview of current EAM integration across available systems is provided in *Table 7*. Electroanatomical mapping is particularly useful in redo procedures or in patients with atypical anatomy. It enhances lesion visualization and, importantly, can be used to assess lesion overlap which is essential for durability of lesion sets.

**Table 7:** EAM integration across PFA systems (alphabetical order)

System	Mapping integration	Limitations
Affera Sphere-9	Proprietary Affera mapping platform with combined mapping/ablation	Interoperability with external 3D systems (CARTO/EnSite) not available at present
Farapulse	Proprietary OPAL HDx mapping platform with catheter orientation and lesion tagging	Simplified visualization; positional accuracy lower than full EAM systems
PulseSelect	Compatible with CARTO, EnSite systems and Affera mapping platform	No native integration; impedance based tracking only, no sensor-based tracking
Varipulse	Fully integrated EAM with lesion tagging	Restricted to CARTO system
Volt	Fully integrated EAM with contact sensing and lesion tagging	At present limited to investigational clinical experience

#### Pulsed field ablation in patients with cardiac implantable electronic devices

Growing clinical experience indicates that PFA can be safely performed in patients with pacemakers and implantable cardioverter-defibrillator (ICD). Larger series have no reported clinically significant interference, lead dislodgement, or device malfunction.^77^ Nonetheless, ablation in immediate proximity to device leads should be avoided, and routine pre- and post-procedural device interrogation is advised. Recent case reports illustrate rare but important exceptions: Nair *et al.* reported permanent failure of a CRT-D and transient software reset with apparent battery depletion of a leadless atrial pacemaker when monopolar PFA was delivered in presumed contact with the SVC coil.^78^ Furthermore, PFA-induced ventricular fibrillation has been described in a patient with a dual-coil ICD, likely mediated by unintended myocardial capture via electromagnetic induction in close proximity to the RV lead.^79^ These observations underscore that while PFA is generally safe in patients with intracardiac devices, lead proximity, and asynchronous PFA delivery may pose a pro-arrhythmic risk. Further data is needed.

#### Pulsed field ablation in patients with left atrial appendage occlusion

**Figure 3:**
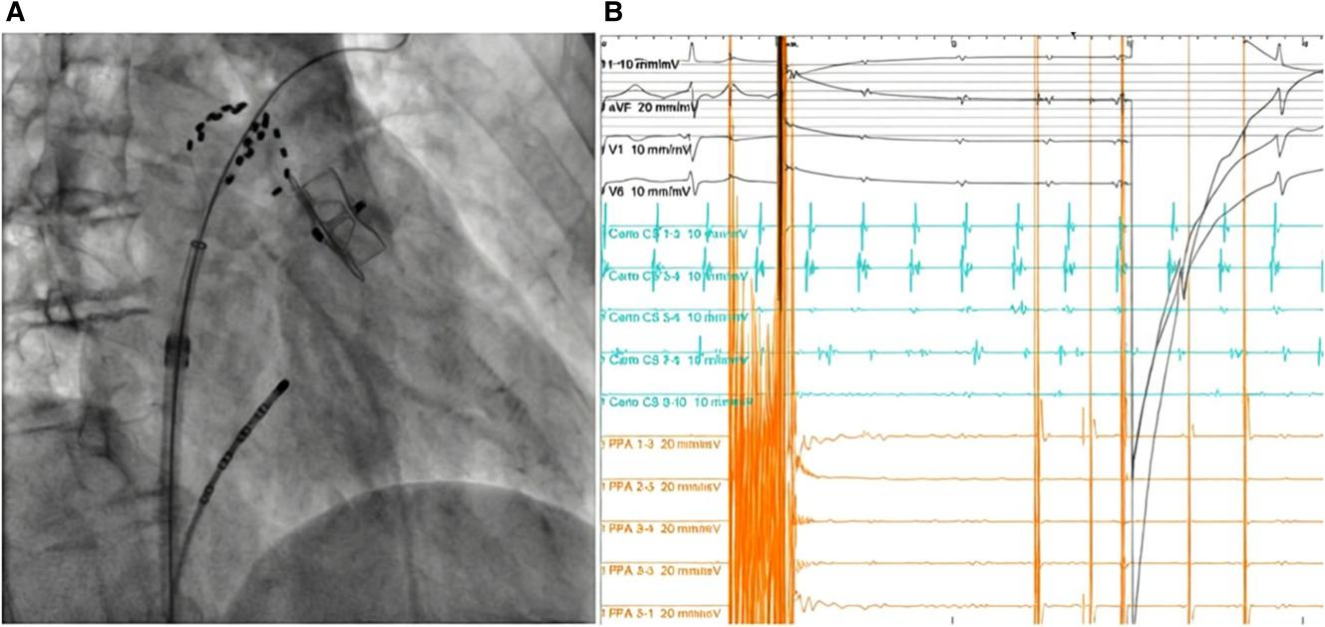
Shown is in RAO the pentaspline PFA catheter and a LAA closure device (Amplatzer Amulet device) with lobe-and-disc design. Real-time EGMs showed artefacts recorded from the PFA catheter electrodes in contact with the adjacent LAA occlusion device (*A*). Multiple attempts to deliver PFA were immediately stopped by the system due to overlapping among the electrodes of the PFA catheter and the disk of the Amulet. Repositioning of the PFA catheter allowed antral PFA and applications were ultimately possible (*B*).

The combination of PFA and LAA occlusion is becoming increasingly relevant. Endocardial plug-type occlusion systems and surgical clip-based closure techniques appear compatible with PFA, whereas lobe-and-disk occluders may interfere with effective energy delivery (*Figure 3*).^80^

In a multicentre series, evaluating PFA performed after prior LAAO, one-third of patients with a lobe-and-disk occluder (such as Amplatzer Amulet, Abbott) experienced aborted applications at left-sided veins due to device proximity, while plug-type occlusion system patients (such as Watchman, Boston Scientific) showed no interference.^80^ The mechanism is geometric, related to disk protrusion into the PV antrum, leading to distortion of the local electric field and triggering system safety interruption, which may require catheter repositioning or in rare circumstances even supplemental RF ablation. Whenever possible, PFA should precede device implantation; if performed after LAAO, device type, and anatomy should be carefully evaluated.

#### Concomitant pulsed field ablation and left atrial appendage occlusion

Combined procedures of PFA and LAAO are feasible. However, whether they should be done routinely is controversial. The OPTION trial demonstrated that LAAO after AF ablation was associated with a significantly lower risk of non–procedure-related major or clinically relevant non-major bleeding compared with oral anticoagulation, but was not superior for major bleeding, which was not a prespecified superiority endpoint. The OPTION trial met its prespecified non-inferiority endpoint for the composite of stroke, systemic embolism, or death. Importantly, it was not designed to assess superiority for individual thromboembolic endpoints.^80,81^ The limited currently available evidence supports an individualized approach, and combined procedures of AF ablation using PFA and LAAO could be evaluated in experienced centres for carefully selected patients, even though guideline-level recommendations are not yet established.

#### Pulsed field ablation in patients with other intracardiac devices

Case reports and newer series show safe outcomes of PFA in patients with ASD/PFO occluders, surgical LAA exclusion systems (AtriClip), and prosthetic heart valves.^82^ While no major device dysfunctions have been reported, risks include arcing, electromagnetic interference, and difficulty delivering energy adjacent to metallic valve components. Therefore, PFA if considered in such patients should be performed with careful catheter positioning, avoiding close proximity and direct contact, and being prepared to use alternate lesion strategies where needed.

### Step-by-step procedure

#### Vascular access

Vascular access is via standard femoral venous puncture. Ultrasound guidance reduces vascular complications.^83^ Anticoagulation is usually initiated pre-transseptal puncture, with ACT maintained ≥300–350 s during the procedure, however, for some systems, an ACT ≥ 350 s is required before catheter manipulation in the left atrium. Use of ultrasound-guided femoral access and uninterrupted anticoagulation is advised.^46^

#### Choice of guidewire

A standard J-tipped guidewire is generally sufficient for over-the-wire devices to advance sheaths into the left atrium and is associated with a favourable safety profile. Extra-stiff wires can provide additional support in patients with tortuous anatomy but increase the risk of perforation. Recent evidence suggests that straight-tip guidewires may cause asymptomatic bronchial bleeding,^84^ whereas J-tip wires do not show this complication.

#### Transseptal puncture

Transseptal puncture remains a key step. Although a posterior puncture is commonly performed, in cases without left atrial enlargement an anterior puncture may facilitate access to the right inferior PV, particularly with certain systems. TSP is often performed using a standard transseptal sheath that is then exchanged over the wire for a dedicated PFA sheath. An over-the-needle TSP directly with the dedicated PFA delivery sheath has been described in a retrospective single-centre experience of 100 patients (‘zero-exchange’).^85^ Dedicated integrated systems with RF wires or short deployable needles, which fit into the larger sheath, also allow puncture in a ‘*zero-exchange*’ workflow. Advanced imaging with ICE or transoesophageal echocardiography (TEE) can further enhance safety, particularly in patients with thick septa or prior closure devices. In practice, TSP should be tailored to the platform and patient anatomy, with ICE advised in challenging cases. Simple measures such as marking the His bundle with a standard diagnostic electrophysiology catheter, marking the aortic root with a guidewire via a retrograde aortic approach, registration of needle pressure, or contrast dye injection, may assist in TSP in some cases.

#### Sheath/catheter exchanges and air embolism prevention

Air embolism is an uncommon but potentially serious complication during PFA. Preventive measures include meticulous de-airing of sheaths and continuous saline flushing. Modern ‘*zero-exchange*’ approaches, available with different platforms, further reduce the risk of air ingress. In addition, ‘zero-exchange’ designs streamline the procedure, shorten fluoroscopy and left atrial dwell time, and may lower embolic risk. The impact of sedation type on air embolism is unknown and requires further research; limited data suggest that negative left atrial pressures, more commonly encountered during deep or conscious sedation than during positive-pressure ventilation under GA, may facilitate air ingress through open haemostasis valves, although the clinical relevance remains to be defined.

#### Vagal response during ablation

Vagal reflexes, including bradycardia, atrioventricular block, or transient asystole, are common during PFA, particularly at the left superior PV. Reported incidence ranges from 30 to 70%, depending on the definition and patient cohort.^86^ Most events are self-limited; atropine (1.0 mg) or, rarely, temporary pacing may be required. Although prophylactic parasympatholytics (e.g. atropine or glycopyrrolate) may reduce the need for backup pacing, their use is contraindicated in patients with glaucoma or benign prostatic hyperplasia owing to the risk of post-procedural urinary retention. In these patients, right ventricular backup pacing may represent a safer alternative. Recently, a RSPV-first ablation strategy has been proposed to mitigate vagal reactions, reducing their occurrence from 78 to 13% compared with an LSPV-first approach.^87^ These autonomic effects are likely due to transient stimulation of the ganglionated plexi (GPs). In summary, vagal responses are frequent and benign; atropine, glycopyrrolate, or pacing may be appropriate if clinically significant.

#### Assessment of catheter–tissue contact

Effective PFA requires stable catheter positioning at the PV antrum.^59^ With pentaspline catheters, coaxial alignment and avoidance of deep catheter positioning within the PV are critical, as inadequate contact may predispose to reconnection. Surrogates for assessing contact include tactile feedback, fluoroscopic spline or circular indentation or ICE, which provides real-time confirmation of ostial positioning. Impedance-based markers integrated into EAM can enable objective contact assessment and have been proven to be useful in an *in vivo* study resulting in significantly larger and more durable lesions, with 100% transmurality when good contact was achieved.^88^ These findings underscore that, despite PFA's field-based mechanism, adequate catheter–tissue proximity remains crucial for durable circumferential lesion formation. In contrast to RFA, PFA is less forgiving with respect to intermittent or suboptimal contact, as its ultra-short pulse delivery lacks thermal latency; consequently, adequate catheter–tissue proximity must be present at the exact moment of energy application to achieve irreversible lesion formation. Moreover, field-induced myocardial or phrenic nerve capture may cause transient catheter instability and could contribute to reversible lesion formation and reconnection. In summary, PFA contact assessment relies on coaxial antral positioning using fluoroscopy, ICE imaging, and increasingly EAM-based tools based on real-time impedance measurements. Future and newly developed integrated contact feedback may further improve durability and workflow standardization.

#### Pulsed field ablation dosing strategies

Pulsed field ablation dosing strategies are currently based largely on manufacturer recommendations, typically defining a minimum number of applications per PV or vein pair. However, the scientific evidence supporting these dosing thresholds is limited, and available clinical studies have not demonstrated a clear association between application number and long-term lesion durability.^89^ It remains unclear whether adjunctive imaging or mapping tools improve durability beyond what could be achieved with additional applications alone. Any escalation in dosing must be balanced against safety considerations. The randomized DOPPIO trial (NCT07021313) is specifically designed to evaluate whether a higher and more targeted number of PFA applications can improve arrhythmia-free survival without compromising procedural safety.

#### Angiographic projections

Standard angiographic projections are typically left anterior oblique (LAO) for the left PVs and right anterior oblique (RAO) for the right PVs, with both views aiding catheter orientation and minimizing the risk of advancing the catheter too deeply into the PV. The use of biplane imaging may be helpful.

#### Role of intracardiac echocardiography/zero-fluoroscopy workflow

**Figure 4:**
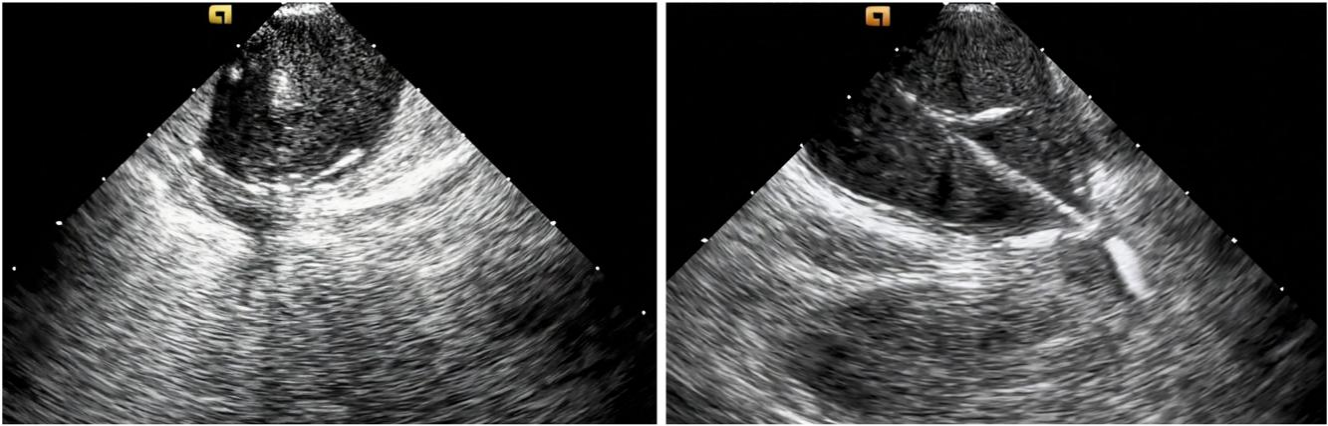
Visualization of the pentaspline PFA catheter by ICE. The pentaspline PFA catheter is deployed in the flower configuration in the right inferior pulmonary vein (left) and in the basket configuration in the left inferior pulmonary vein (right). ICE allows visualization of catheter–tissue contact. This also ensures that the PFA catheter remains at the ostium and not inside the pulmonary vein.

Intracardiac echocardiography offers real-time visualization of cardiac anatomy. Intracardiac echocardiography has been proven to be not inferior to TEE in excluding LAA thrombus.^90^ Intracardiac echocardiography allows for a safe transseptal puncture, including in challenging anatomic variants as lipomatous hypertrophy of the interatrial septum, atrial septal aneurysm, or in patients with prior surgical or PFO or ASD repair.^91^ In AF ablation using RF energy, ICE has been shown to reduce radiation exposure, shorten the overall procedure duration, and enhance safety by facilitating early recognition of acute complications, such as pericardial effusion.^91–93^ Intracardiac echocardiography enables confirmation of tissue contact and real-time monitoring of the PFA catheter position relative to the PV ostium (*Figure 4*). In some reports, the use of ICE has been shown to improve PVI durability, achieve better success rates, and significantly reduce reconnection rates with the pentaspline catheter.^60,94^ On the other hand, ICE remains an invasive procedure that requires additional skills, venous access, and catheter manipulation, and it carries potential risks such as vascular injury and cardiac perforation.

Intracardiac echocardiography can assist transseptal puncture, verify catheter positioning, contact, and detect complications such as effusion. Although not mandatory, it may enhance safety in high-risk patients. In centres pursuing a zero-fluoroscopy workflow, ICE also facilitates real-time anatomical guidance that obviates the need for radiographic imaging.

#### Troubleshooting (energy interruption, electrical arcing)

Automatic interruption of energy delivery may occur across PFA platforms if the system detects unsafe geometry, abnormal impedance behaviour, or potential interaction with metallic implants. Electrical arcing remains rare but has been described particularly when electrodes are in direct contact with each other or adjacent metal. Prevention strategies should focus on optimized catheter contact, flushing, and avoiding proximity and contact with metallic devices like occluders and artificial valves. In most cases, energy delivery resumes after correction of catheter geometry and stable antral alignment. *Table 8* summarizes typical triggering conditions across different catheter concepts.

**Table 8:** Examples of conditions associated with interruption of PFA energy delivery

Catheter concept (generic)	Typical triggering condition	Practical implication
Multi-spline loop catheters (incl. pentaspline)	Excessive proximity of individual splines/loss of stable antral alignment/electromagnetic interaction with adjacent metallic implants	If splines are touching or excessively close, energy delivery is inhibited
Variable-loop catheters	Loop diameter reduced below the validated geometric range (electrode–electrode spacing too small)	Generators will typically refuse to deliver energy until the loop diameter is increased
Focal PFA tip/lattice-tip catheters	Abrupt temperature overshoot or impedance rise when the tip is wedged in a tight angle or excessive forward force is applied	Gentle retraction or slight rotation to a less constrained angle often restores safe energy delivery
Balloon-based multielectrode systems	Partial loss of wall contact, or artefact from air/saline/blood mixing around the electrodes	Correction of balloon position and elimination of air typically restores energy delivery

Only interference with a lobe-and-disk LAA occluder has been formally described as a cause of interrupted delivery;^95^ all other scenarios reflect generic safety logic of contemporary PFA platforms.

### Procedural endpoint definition

Pulsed field ablation leads to rapid attenuation of near-field signals, sometimes complicating their distinction from far-field activity. Accurate electrogram interpretation remains essential, although more challenging after PFA, to confirm PV isolation.^96^ Differences in electrode size and inter-electrode spacing across PFA catheter designs affect near-field vs. far-field discrimination. Smaller electrodes with tighter spacing improve local resolution but may be more susceptible to signal suppression after PFA, whereas larger electrodes with wider spacing increase far-field content and may mask residual conduction. Thus, catheter-specific behaviour needs to be accounted for when interpreting signals after PFA.

Durable entrance and exit block remain universal endpoints; however, strategies vary by platform. While fully mapping integrated systems rely on multipolar mapping after ablation, others may be combined with dedicated 3D-EAM systems^74^ or use pacing manoeuvres. Comparative studies assessing different strategies are lacking. The prognostic role of pacing for PV isolation validation for long-term durability after PFA is less established than after RF ablation.^76^

In thermal ablation, adenosine identifies dormant conduction. With PFA, the yield is low, since even stunned veins with incomplete isolation may not demonstrate dormant conduction.^49^ Similarly, the value of a standardized waiting time is low. Pharmacological testing may be appropriate but is not required in routine PFA. Given the wide variation in lesion delivery strategies across platforms, it is advisable to adhere to manufacturer-recommended energy delivery protocols and to apply only a limited number of additional applications in the presence of residual signals, to avoid unnecessary overtreatment.

### Post-procedural care/same-day discharge protocols

Post-procedural management after PFA differs somewhat from thermal ablation^97^ given its lower incidence of PV stenosis, pericarditis,^98^ PNI, and AEF.^99,100^ Pulsed field ablation–specific complications such as haemolysis leading to acute kidney injury (AKI)^101–103^ or coronary vasospasm have been reported and are discussed separately in Pulsed field ablation related adverse events.^104^ In line with current AF ablation practice, therapeutic anticoagulation should be continued uninterrupted for at least 2 months post-ablation, with subsequent continuation based on CHA₂DS₂-VA risk.^46^ Most centres abandoned a standard proton-pump inhibitor therapy after PFA. Venous vascular closure systems are increasingly used after AF ablation, but data in the PFA era remain limited.^105^ Same-day discharge has been evaluated in prospective registries and shown to be safe in selected patients.^106,107^ Recent data, including an admIRE trial sub-analysis^107^ and an EHRA meta-analysis,^108^ support the safety of same-day discharge after AF ablation when appropriate patient selection and standardized protocols are applied. However, same-day discharge may not be appropriate in patients with procedural complications, significant comorbidities, or those requiring extended monitoring. Further practical advice will be provided in a separate EHRA consensus document. Practical advice regarding procedural workflow in PFA is summarized in *Table 9*.

**Table 9:** Practical advice: procedural workflow in pulsed field ablation

Step	Practical advice
Pre-procedure	Use either general anaesthesia or deep sedation, or in selected platforms/centres conscious sedation; CT/MRI can support planning but is not mandatory.
Electroanatomical mapping	EAM use routinely for first-time PVI is not mandatory; could be employed in redo procedures, atypical anatomy, or when additional lesion sets are planned.
CIED patients	PFA can usually be performed safely in CIED patients using standard precautions: avoid close proximity and direct ablation near leads, and interrogate the device before and after the procedure. However, device–PFA interactions may be platform-dependent and cases of clinically relevant CIED malfunction have been reported; heightened caution and manufacturer-specific guidance are advised, particularly for hybrid RF/PFA systems.
LAAO patients	Be aware of potential arcing with metallic devices. Prefer performing PFA before LAAO implantation; if done after LAAO, proceed with caution. Reserve concomitant PFA + LAAO procedures for selected patients and expert centres.
Vascular access and anticoagulation	Use ultrasound-guided femoral access to reduce complications. Maintain ACT ≥ 300–350 s and continue oral anticoagulation uninterrupted where possible.
Transseptal puncture	Use a posterior puncture as standard, use ICE in difficult cases. Tailor puncture site to system and patient anatomy.
Sheath handling	Apply strict flushing protocols where needed with continuous saline to minimize thromboembolism. ‘Zero-exchange’ workflows can be employed where feasible.
Vagal responses	Anticipate vagal reflexes, especially at LSPV/LIPV. They are usually self-limited; treat clinically relevant episodes with atropine, and use pacing only rarely if needed.
Catheter–tissue contact	Catheter–tissue contact is often inferred rather than directly measured: tactile feedback, fluoroscopic spline indentation, pacing response, ICE, or EAM/impedance-based feedback to confirm antral positioning and adequate proximity.
Validation	Always validate acute entrance (and when appropriate exit) block as the procedural endpoint. Be meticulous with near- vs. far-field discrimination. Pacing and pharmacological testing may be used selectively, acknowledging that evidence for improved prediction of long-term durability is limited.
Post-procedure	Post-procedural management (including anticoagulation duration and rhythm monitoring) should follow contemporary AF ablation guidelines. Monitor for vascular complications, pericardial effusion, and coronary spasm. Same-day discharge is feasible in stable, low-risk patients.

## Efficacy and safety

### Clinical outcomes

#### Randomized controlled trials

To date, there have been five randomized controlled trials comparing PFA to thermal ablation: The Pulsed Field or Conventional Thermal Ablation for Paroxysmal AF trial (ADVENT), the Pulsed Field or Cryoballoon Ablation for Paroxysmal AF trial (SINGLE SHOT CHAMPION) trial, the PFA vs. RFA for the treatment of paroxysmal AF trial (BEAT PAROX-AF), and the PFA using a novel nanosecond biphasic catheter vs. conventional thermal ablation for paroxysmal AF trial (INSIGHT PFA) were performed in patients with paroxysmal AF.^41,42,109^ The Dual-energy lattice-tip ablation system for persistent atrial fibrillation trial (SPHERE Per-AF) was performed in patients with persistent AF.^45^

The key findings of these five trials are summarized in *Table 10*. In aggregate, freedom from atrial tachyarrhythmia recurrence following index ablation after a blanking period was similar between randomized technologies, meeting non-inferiority but not superiority (except for SINGLE SHOT CHAMPION^110^). Similarly, there was no difference in time spent in AF (AF burden), quality of life, or healthcare utilization. Complications were low and not significantly different between randomized technologies, but the use of PFA resulted in shorter procedure times across all studies.

**Table 10:** Randomized controlled trials comparing PFA to thermal ablation

Registries	PF catheter and comparator	PF catheter manufacturer	Patients	Sites and enrolment period	AF type	Endpoint definition	Monitoring strategy	Efficacy endpoint	Safety endpoint	Procedure time
ADVENT^41^	Pentaspline catheter Thermal ablation	Boston Scientific	607	30 (03/2021–06/2022)	PAF— 100.0%	Freedom from treatment failure, AT/AF recurrence, cardioversion, reinitiation of AAD, or reablation	Intermittent (72 h Holter after 6 and 12 months plus weekly TTM)	PFA—73.3% Thermal—71.3%	PFA—2.1% Thermal—1.5%	PFA—106 min Thermal—123 min
SINGLE SHOT CHAMPION^42^	Pentaspline catheter Cryoballoon ablation	Boston Scientific	210	2 (09/2022–11/2023)	PAF—100.0%	Freedom from AT/AF recurrence	Continuous (ICM)	PFA—62.9% CBA—49.3%	PFA—1.0% CBA—1.9%	PFA—55 min CBA—73 min
BEAT-AF	Pentaspline catheter Radiofrequency ablation	Boston Scientific	289	9 (04/2021–01/2024)	PAF—100.0%	Freedom from AT/AF recurrence, reinitiation of AAD, or reablation	Intermittent (24 h Holter after 2, 6, and 12 months plus weekly TTM)	PFA—77.2% RFA—77.6%	PFA—4.8% RFA—7.6%	PFA—56 min RFA—95 min
INSIGHT PFA	Lotus catheter Radiofrequency ablation	Insight Lifetech	287	9 (09/2023–06/2024)	PAF—100.0%	Freedom from AT/AF recurrence, reinitiation of AAD	Intermittent (24 h Holter after 6 and 12 months)	PFA—66.7% RFA—67.4%%	PFA—18.3% RFA—17.9%	PFA—99 min RFA—113 min
Sphere persAF^45^	Lattice tip dual-energy catheter Radiofrequency ablation	Medtronic	420	23 (12/2021–12/2022)	PsAF—100.0%	Freedom from treatment failure, AT/AF recurrence, cardioversion, reinitiation of AAD, or reablation	Intermittent (24 h Holter after 6 and 12 months)	Lattice tip—73.8% RFA—65.8%	Lattice tip—1.4% RFA—1.0%	Lattice tip—101 min RFA—126 min

AF, atrial fibrillation; AT, atrial tachyarrhythmia; PAF, paroxysmal atrial fibrillation; PFA, pulsed field ablation; CBA, cryoballoon ablation; RBA, radiofrequency ablation; PsAF, persistent atrial fibrillation; ICM, implantable cardiac monitor; AAD, antiarrhythmic drugs.

#### Single-arm registries

Several pivotal trials for regulatory approval of the different PFA devices have been performed. Across these pivotal regulatory studies, efficacy outcomes were assessed using protocol-defined rhythm monitoring in accordance with CE mark and FDA requirements, including scheduled follow-up visits with systematic ECG and ambulatory monitoring beyond symptom-driven assessment. The admIRE and inspire studies evaluated an irrigated variable-loop circular PFA catheter, the PULSED-AF study evaluated an over-the-wire loop PFA catheter, the SmartfIRE study a dual-energy focal catheter and the Omny-IRE a large-tip focal, multielectrode PFA catheter, the IMPULSE, PEFCAT, PEFCAT II and ADVANTAGE studies evaluated the pentaspline catheter and the VOLT CE mark study^36^ reported on a basket-in-balloon PFA catheter (*Table 11*).^44,52,60,100,111–115^ Furthermore, large single-arm registries and studies have reported on safety and efficacy outcomes with the pentaspline catheter (EU-PORIA, MANIFEST, MANIFEST 17k, FARADISE) (*Table 12*).^99,117–119^ And finally, several non-randomized trials compared PVI outcomes after PFA and thermal energies. In aggregate, these studies reported a 1-year freedom from recurrent atrial tachyarrhythmia ranging from 55 to 80% [paroxysmal 66–82%, vs. persistent 55–71%], with no statistically significant difference in the rate of recurrence (RR 0.85; 95% CI 0.64–1.14; *P* = 0.29).^120^

**Table 11:** Pivotal trials for regulatory approval

Studies	PF catheter	Manufacturer	Patients	Sites and enrolment period	AF type	Acute post-procedural success	Follow-up	Freedom from AF/AT	Major adverse events^a^, *n* (%)	PF-specific adverse events, *n* (%)
inspIRE^111^	Variable-loop circular catheter	Biosense Webster	272	13 (08/2020–05/2022)	PAF—100.0%	97.1%	12 months	70.9%	Total: 0 Tamponade—0 Death—0 Stroke—0 Major vascular—0	Coronary spasm—NR Oesophageal fistula—0 PV stenosis—0 Persistent PNI—0 Haemolysis—NR
AdmIRE^112^	Variable-loop circular catheter	Biosense Webster	277	30 (04/2022–11/2022)	PAF—100.0%	100.0%	12 months	75.4%	Total: 8 (2.9%) Tamponade—3 (1.1%) Death—0 Stroke—2 (0.7%) Major vascular—2 (0.7%)	Coronary spasm—0 Oesophageal fistula—0 PV Stenosis—0 Persistent PNI—0 Haemolysis—0
PULSED AF^100^	Circular array catheter	Medtronic	300	41 (03/2021–11/2021)	PAF—50.0% PsAF—50.0%	100.0%	12 months	PAF—66.2% PsAF—55.1%	Total: 2 (0.7%) Tamponade—1 (0.3%) Death—0 Stroke—1 (0.3%) Major vascular—0	Coronary spasm—NR Oesophageal fistula—0 PV stenosis—0 Persistent PNI—0 Haemolysis—NR
Sphere-360 European Study^116^	Large-lattice circumferential catheter	Medtronic	100	3 (NR—NR)	PAF—100.0%	100.0%	12 months	82.0%	Total: 0 Tamponade—0 Death—0 Stroke/TIA—NA Major vascular—0	Coronary spasm—NR Oesophageal fistula—0 PV stenosis—0 Persistent PNI—0 Haemolysis—NR
IMPULSE, PEFCAT, and PEFCATII^60^	Pentaspline catheter Focal catheter for CTI	Boston Scientific	121	3 (NR—NR)	PAF—100.0%	100.0%	12 months	79.3%^b^	Total: 2 (1.6%) Tamponade—1 (0.8%) Death—0 Stroke—1 (0.8%) Major vascular—1 (0.8%)	Coronary spasm—NR Oesophageal fistula—0 PV stenosis—0 Persistent PNI—0 Haemolysis—NR
VOLT CE MARK Study^52^	Balloon-in-basket Catheter	Abbott	150	11 (11/2023–03/2024)	PAF—70.5% PsAF—29.5%	98.1%	6 months	PAF—88.2% PsAF—76.7%	Total: 4 (2.7%) Tamponade—1 (0.7%) Death—0 Stroke—1 (0.7%) Major vascular—2 (1.4%)	Coronary spasm—0 Oesophageal fistula—0 PV stenosis—0 Persistent PNI—0 Haemolysis—NR
SPHERE Per-AF^45^	Lattice-tip catheter	Medtronic	420	20 (12/2021–12/2022)	PsAF—100.0%	NR	12 months	73.5%	Total: 0 (0.0%) Tamponade—0 (0.0%) Death—0 (0.0%) Stroke/TIA—0 Major vascular—0	Coronary spasm—NR Oesophageal fistula—0 PV stenosis—0 Persistent PNI—0 Haemolysis—NR
ADVANTAGE AF^44^	Focal-linear catheter	Medtronic	255	29 (10/2023–03/2024)	PsAF—100.0%	99.6%	12 months	77.8%^c^	Total: 6 (2.4%) Tamponade—1 (0.4%) Death—1 (0.4%) Stroke/TIA—3 (1.2%) Major vascular—1 (0.4%)	Coronary spasm—NR Oesophageal fistula—NR PV stenosis—NR Persistent PNI—NR Haemolysis—NR

AF, atrial fibrillation; AT, atrial tachyarrhythmia; CTI, cavotricuspid isthmus; PAF, paroxysmal atrial fibrillation; PF, pulsed-field; PNI, phrenic nerve injury; PsAF, persistent atrial fibrillation; PV, pulmonary vein; NR, not reported; TIA, transient ischaemic attack.

^a^Major adverse events as defined in each individual study. Not all subcategories of major adverse events are described.

^b^Refers to single procedure (i.e. with no ablation during remapping or otherwise).

^c^Pulmonary vein and posterior wall isolation (100%) and 55.3% had concomitant cavotricuspid isthmus ablation.

**Table 12:** Large multicentre post-approval PFA registries

Registries	PF catheter	Manufacturer	Patients	Sites and enrolment Period	AF type	Acute post-procedural success	Follow-up	Freedom from AF/AT^a^	Major adverse events^b^ *n* (%)	PF-specific adverse events *n* (%)
MANIFEST-PF^99^	Pentaspline catheter	Boston Scientific	1568	24 (03/2021–05/2022)	PAF—65.0% PsAF—32.0%	99.2%	12 months	PAF—81.6% PsAF—71.5%	Total: 30 (1.9%) Tamponade—18 (1.1%) Death—1 (0.06%) Stroke—6 (0.4%) Major vascular—2 (0.1%)	Coronary spasm—2 (0.1%) Oesophageal fistula—0 PV stenosis—0 Persistent PNI—0 Haemolysis—NR
MANIFEST-17k^118^	Pentaspline catheter	Boston Scientific	17 642	106 (01–03/2022–06/2023)	PAF—57.8% PsAF—35.2%	NR	Peri-procedural Only	N/A	Total: 173 (0.9%) Tamponade—63 (0.36%) Death—5 (0.03%) Stroke—22 (0.12%) Major vascular—53 (0.3%)	Coronary spasm—25 (0.14%) Oesophageal fistula—0 PV stenosis—0 Persistent PNI—0 Haemolysis—6 (0.03%)
EU-PORIA^117^	Pentaspline catheter	Boston Scientific	1233	7 (03/2021–05/2022)	PAF—60.2% PsAF—39.8%	99.96%	12 months	PAF—80.0% PsAF—66.0%	Total: 21 (1.7%) Tamponade—14 (1.1%) Death—0 Stroke—5 (0.41%)	Coronary spasm—1 (0.1%) Oesophageal fistula—0 PV stenosis—0 Persistent PNI—0 Haemolysis—NR

AF, atrial fibrillation; AT, atrial tachyarrhythmia; PAF, paroxysmal atrial fibrillation; PF, pulsed-field; PsAF, persistent atrial fibrillation; PNI, phrenic nerve injury; PV, pulmonary vein; NA, not available; NR, not reported.

^a^Defined as AF/AT > 30 s. Typically follow-up at 3, 6, and 12 months and additional Holter monitors after 90 days of blanking period.

^b^Major adverse events as defined in each individual study. Not all subcategories of major adverse events are described.

#### Blanking period

For thermal ablation modalities, the duration of the post-procedure ‘blanking period’ was recently shortened from 3 months to 8 weeks.^46^ Pulsed field ablation has a different inflammatory profile, and there is emerging evidence that acute peri-procedural changes are less profound than with thermal ablation. In the SINGLE SHOT CHAMPION trial, a significantly lower rate of recurrence was observed for PFA in the blanking period using continuous rhythm monitoring (38.1% vs. 58.1%).^42^ A secondary analysis of the PULSED AF trial showed that early recurrence of atrial tachyarrhythmia within the first 3 months after ablation was significantly associated with an increased risk of late recurrence.^121^ This is consistent with two other studies indicating that recurrences occurring in post-procedure months 2 and 3 were universally associated with later treatment failure.^122,123^ As such, a blanking period of just 1 month after PFA may be reasonable for clinical decision-making, whereas the use of a blanking period in clinical trials involving PFA should be reconsidered moving forward.^2^

### Occurrence and management of adverse events

**Figure 5:**
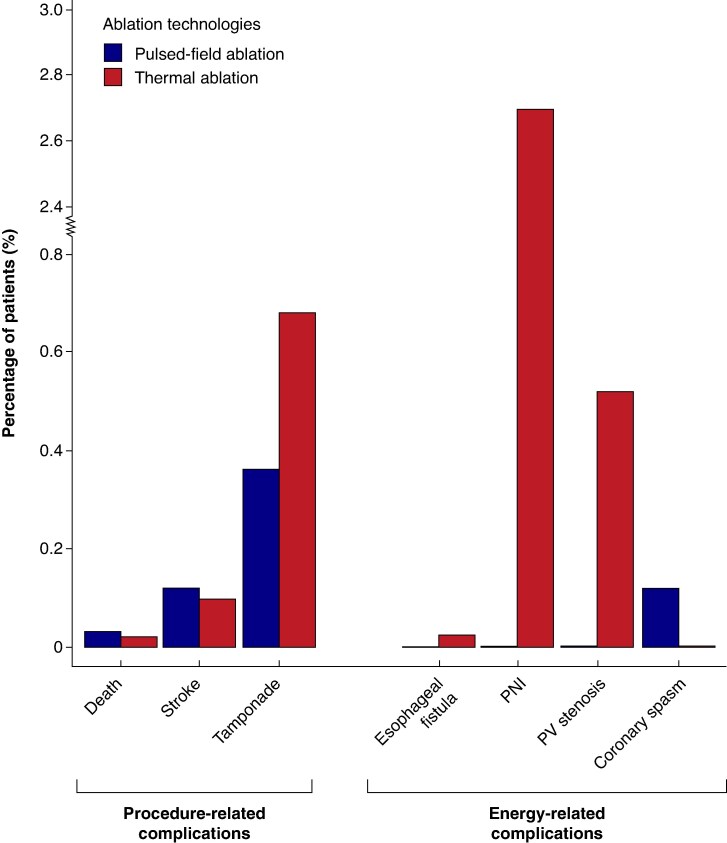
Summary of procedure-related and energy-related complications associated with thermal and pulsed field ablation, including: death^97,118^, stroke^97,118^, tamponade^97,118^, esophageal fistula^39,97,118^, phrenic nerve injury (PNI)^97^, pulmonary vein (PV) stenosis^97^, coronary spasm.^97^

After the recent introduction of PFA into clinical practice, an overall favourable safety profile with some occasional but unexpected adverse events have been reported (*Figure 5*).^124^ Although PFA was initially considered cardioselective and largely contact force independent, accumulating evidence indicates that PFA is tissue-selective, contact-dependent, minimally thermal, and voltage-dependent, with safety profiles that may vary across different PFA platforms and clinical applications. Appropriate specialized EP training remains key to maintain the quality and safety of PFA procedures (see also Section Training and education).

#### Pulsed field ablation–related adverse events

##### Haemolysis/acute kidney injury

Post-PFA haemolysis and occasional AKI has been observed, correlated with high number of applications, particularly with higher-voltage systems.^125,126^ The incidence appears to vary across PFA platforms, likely reflecting differences in waveform characteristics, voltage amplitude, and catheter design. The risk of AKI may be reduced with optimal pre-ablation hydration.^102,113–115,118^ Based on available evidence, the number of PFA applications should be chosen mindfully and pre-ablation hydration may be appropriate, especially in patients with pre-existing chronic kidney disease.^102,127^

##### Phrenic nerve injury

Phrenic nerve injury, a known complication of thermal AF ablation, was reported to be transient in 0.006% and permanent in none (0%) in the MANIFEST-17K study.^118^ Importantly, phrenic nerve capture during PFA pulse delivery should be distinguished from nerve injury, as these represent different electrophysiological mechanisms.

##### Coronary injury/drug protocols for prevention of vasospasm

Proximity-related coronary vasospasm was widely documented during PFA for cavotricuspid and mitral isthmus ablation, initially when using the pentaspline PFA catheter,^104^ but later similarly with several other PFA devices, most likely indicating a voltage/field strength dependence. The likelihood and severity of vasospasm may therefore vary across PFA systems, reflecting differences in energy delivery and electric field distribution. Spasm typically develops within seconds to a few minutes after PFA, although delayed onset has also been reported.^128^ These observations are clinically relevant, particularly in the context of streamlined procedural workflows and increasing use of same-day discharge strategies after AF ablation. It can be largely attenuated by administration of nitroglycerine either for treatment (1–2 mg intracoronary) or as prophylaxis (intracoronary 1 mg or intravenous 1–2 mg).^104^ Coronary vasospasm observed after PFA may relate to transient functional effects on the coronary vasculature, including endothelial or smooth muscle electroporation, autonomic influences, or circulating vasoactive factors, although the underlying mechanisms remain incompletely understood.^104^ Given the potential for both acute and delayed vasospasm, careful intra-procedural management and early post-procedural monitoring may be warranted in selected patients. Also, the long-term effect of PFA on coronary arteries is currently unknown.^129^ Further systematic investigation will be important to better define the incidence, mechanisms, and clinical implications of coronary vasospasm across different PFA platforms.

##### Pulmonary vein stenosis

Although severe PV stenosis is rare, mild to moderate narrowing of PVs has been observed frequently in patients undergoing thermal ablations but was not observed with PFA-only devices.^40,109^ However, systematic assessment for subclinical PV narrowing is not routinely performed in real-word practice.

##### Oesophageal events

Oesophageal injury is a rare but often fatal complication of thermal ablation, occurring with an incidence of 0.025% and a mortality of 66%. To date, not a single case of oesophageal fistula has been reported despite the use of PFA in >500 000 patients worldwide.^38,118,130^

##### Conduction system disturbances

Collateral injury to the AV node or sinus node can occur using thermal energy. While cases of occasional and often transient AV block or sinus node dysfunction have been reported after ablation of atrial flutter (AFl) or isolation of the superior vena cava using PFA, this scenario is rare.^55,131^ The full impact of PFA on the conduction system however is yet to be explored.

##### Pericarditis

The incidence of acute pericarditis following pulsed field AF ablation ranges from 0 to 0.3% in recent multinational registries and prospective studies.^41,100,117,118^ Studies specifically investigating pericarditis rates report a broader range (0–4.4%), depending on the definition used (e.g. with or without ESC criteria).^98^ Similar variability is observed when compared to thermal AF ablation procedures, where reported incidences range from 0 to 10.2%.^41,132,133^

##### Effect on left atrial function

During the acute phase following PFA-PVI, most studies report a reduction in markers of left atrial (LA) compliance and contractile function.^134–136^ This is observed both after PVI alone and following more extensive LA ablation, likely reflecting transient LA-stunning.^134–136^ In the long-term (chronic phase), markers of LA-compliance typically return to baseline,^134–137^ although persistent impairment of LA contractile function at 3 months after PFA-PVI despite recovery of reservoir function has been described.^119^ In contrast, RF-PVI with extensive LA ablation leads to a long-term decline in LA-compliance markers.^138^

#### Non-pulsed field ablation–related events

##### Pericardial tamponade

The incidence of pericardial tamponade during PFA for PVI ranges from 0 to 1.1% in prospective randomized studies and large registries, which is similar compared to thermal ablation.^41,42,45,97,100,117–119,139^

##### Stroke/transient ischaemic attack/cerebral emboli

The incidence of stroke and transient ischaemic attacks (TIA) during PFA for PVI has been reported at 0–1% and 0–0.3%, respectively, in recent trials.^41,42,100,117,118^ Large registries of thermal PVI procedures report similar combined stroke/TIA rates ranging from 0.1 to 1%. Notably, post-market evaluation of a variable-loop circular PFA catheter (Varipulse, Biosense Webster) led to updated use instructions following a numerically higher early stroke/TIA rate. A recent meta-analysis reported silent cerebral event (SCE) rates of 14% for PFA, 18% for RF, and 21% for cryoballoon ablation procedures.^140^ Importantly, SCE observed after PFA should not be attributed solely to the electroporation mechanism. Available evidence suggests a multifactorial process that may involve air embolism during catheter exchanges, thrombus formation on sheaths or electrodes, transient hypotension, and peri-procedural anticoagulation management. Moreover, emerging data indicate substantial variability between PFA platforms, suggesting that catheter design, waveform characteristics, and energy delivery may influence cerebral embolic risk.^141^

##### Vascular access complications

Vascular access complications in PFA procedures occurred in 0–2.2% of cases.^41,100,117,118^ The use of ultrasound guidance in patients of the Manifest-17K cohort was associated with a two thirds reduction in access site complications.^118^

##### Death

Reported mortality rates for PFA-based interventions range from 0 to 0.3% and are most commonly attributable to cardiac tamponade or stroke.^41,100,117,118^ By comparison, registries of thermal AF ablation report mortality rates between 0.02 and 0.46%.^97,142^ Rare cases of delayed malignant arrhythmias, myocardial ischaemia, and sudden cardiac death after PFA have been reported, particularly following ablation beyond PVI. Although causality remains unproven and incidence appears very low, these observations highlight the need for vigilance and further mechanistic study.^143^

### Autonomic nervous system effects

Pulsed field (PF) energy interacts with the cardiac autonomic nervous system—specifically the GPs—in ways that differ from thermal ablation. Acutely, the application of PF-impulses during PVI often triggers vagal responses, likely due to intense stimulation of nearby GPs, particularly during LSPV ablation.^144^ Initiating PFA-PVI at the RSPV—possibly ‘suppressing’ the anterior right GP—can reduce vagal response during subsequent ablation, e.g. of the LSPV.^87^ Chronically, durable neural injury however appears minimal. Unlike RF or cryo-PVI, PFA-PVI does not lead to a significant post-procedural increase in heart rate or neuronal injury markers (e.g. SF100B).^144–146^

### Long-term follow-up data

Outcome data beyond 1 year after AF ablation using PFA is currently lacking. Data on the durable effect on arrhythmia suppression after PFA-PVI and the prevention of progression from paroxysmal AF to persistent AF will be important.

## Training and education

**Figure 6:**
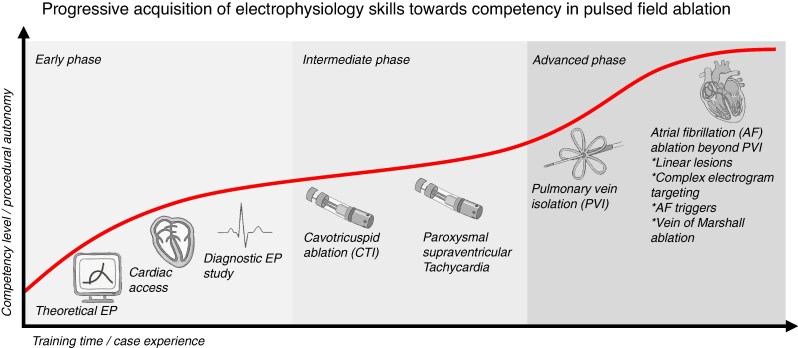
Progressive acquisition of electrophysiology competencies towards PFA proficiency. The learning curve illustrates the sequential development of key procedural skills required to achieve competency in PFA. Foundational competencies include use of EP laboratory equipment, vascular and cardiac access, and performance of diagnostic EP studies. Intermediate competencies encompass CTI and non-CTI atrial tachycardia ablations. Advanced competencies involve PVI and atrial fibrillation ablation beyond PVI, including linear lesions, complex electrogram targeting, and vein of Marshall ablation. Mastery of these domains culminates in independent, safe, and efficient performance of PFA procedures.

Pulsed field ablation offers efficiency and improved safety when compared with thermal ablation. These features have generated enthusiasm across specialties. Specialized education in EP brings essential expertise in arrhythmia mechanisms, mapping, and intracardiac signal interpretation, competencies that allow comprehensive AF care. In line with these principles, PFA procedures should be performed under the responsibility of physicians holding recognized electrophysiology certification, such as the EHRA EP certification, which delineates the requisite expertise for independent practice (*Figure 6*). In addition, centres adopting PFA should ensure structured, centre-specific education of the entire electrophysiology team and actively encourage participation in nationwide procedural registries to enable continuous quality assurance, benchmarking, and transparent outcome monitoring.

### Structured training pathway

Effective PFA training should integrate both theoretical and practical competencies. Didactic instruction should include the biophysical principles of electroporation, tissue specificity, safety margins, and device-dependent characteristics.^147^ Equally important is the acquisition of procedural knowledge such as patient selection, pre-procedural planning, and peri-procedural management. Hands-on training should ideally begin with simulators or animal models before progressing to supervised clinical practice under the guidance of experienced PFA operators. Mentorship and proctoring during the early learning phase prevent unsafe extrapolation from RF or cryoballoon ablation.^147^ What distinguishes PFA from prior innovations is not only its promise of efficiency but also the depth of electrophysiological understanding required to harness it safely. Competence in mapping, interpretation of intracardiac signals, and the management of complex atrial arrhythmias remain fundamental.

### Learning curve and competency milestones

Early experiences with PFA suggest that acute PVI can be achieved with high reproducibility after relatively few procedures.^148–150^ Registry data indicate similar outcomes between junior and senior electrophysiologists.^117^ These findings make PFA appear more accessible than point-by-point RF ablation. Yet lessons from cryoballoon adoption caution against oversimplification: inappropriate case selection, incomplete understanding of atrial anatomy, and neglecting electrophysiological endpoints may compromise results despite apparently straightforward workflows.^151^

**Figure 7:**
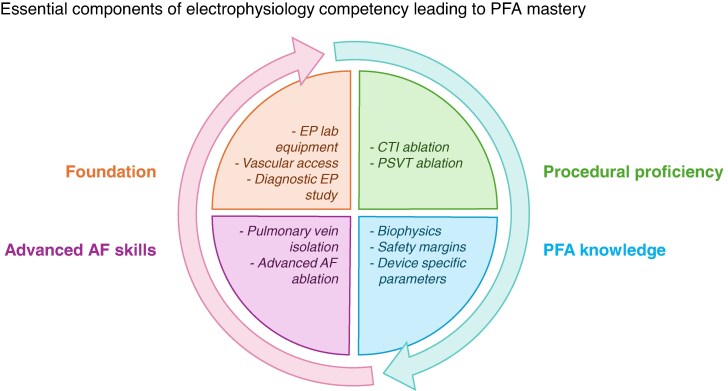
Essential components of electrophysiology competency leading to PFA mastery.

Thus, the real learning curve is broader—achieving competence not only in PVI but also in ablation beyond the veins, repeat procedures, and non-PV arrhythmias (*Table 13* and *Figure 7*). A structured pathway with exposure to different catheter designs and system platforms (balloon-based and point-by-point)^52^ and to anatomically challenging cases is essential (see Section Procedural workflow).^153,154^ Structured case numbers, milestone-based assessments, and continuous procedural feedback should be incorporated into training.^152^

**Table 13:** Objective structured assessment of technical skills for AF ablation using PFA (modified from EHRA core curriculum)^152^

1. Safe use of EP laboratory equipment relevant to PFA, including fluoroscopy and/or electroanatomical mapping as required by the specific platform; understanding of PFA-specific biophysics (irreversible electroporation, tissue selectivity, pulse characteristics) and how these differ from thermal energy.
2. Venous access, transseptal puncture, anticoagulation management, and imaging guidance (fluoroscopy, ICE where indicated) tailored to PFA system requirements.
3. System-specific catheter handling (single-shot, balloon-based, lattice, or focal PFA), correct antral positioning, avoidance of deep or ostial deployment, and recognition of suboptimal positioning requiring repositioning or repeat applications.
4. Perform PVI using PFA, including anatomical understanding of the PV antra, appropriate application strategies, management of system-specific phenomena (e.g. phrenic nerve capture, extracardiac muscle stimulation), and recognition and management of acute complications.
5. Verification of *acute* pulmonary vein entrance (and when appropriate exit) block, with correct near- vs. far-field signal interpretation; understanding the limitations of acute assessment in predicting long-term durability.
6. Recognition and management of vagal responses, transient conduction disturbances, coronary spasm, phrenic nerve capture, cough or diaphragmatic contraction, and platform-specific interactions with intracardiac devices or metallic implants.
7. System- and indication-specific use of PFA for additional lesion sets (e.g. posterior wall, superior vena cava, linear lesions), including appropriate patient selection, anatomical considerations, and safety limitations specific to PFA.

The training requirements and skills of the latter are detailed in the EHRA Core Curriculum document.^152^

### Integration into electrophysiology fellowship programmes

Training in PFA should occur within established centres of excellence in cardiac electrophysiology. High procedural volume, access to advanced imaging and mapping tools, and a culture of mentorship create the optimal environment for structured learning. Embedding PFA into electrophysiology fellowship curricula ensures early exposure and integration into a comprehensive procedural repertoire. In this context, the EHRA Educational Pathway provides a structured framework for competency-based training in AF ablation, with AF-specific courses embedded at Level II and formal knowledge assessment through the EHRA electrophysiology examination, which is strongly advised as part of professional development.

### Non-technical skills and professional standards

Alongside procedural competence, modern training must address non-technical skills. These include clinical judgment for patient selection, recognition of contraindications, interdisciplinary communication, and the capacity for patient-centred decision-making. The recent consensus document on catheter and surgical ablation^46^ emphasizes the importance of institutional readiness, team-based training, and longitudinal follow-up. Similarly, the atrial tachycardia consensus document (SMART-AT roadmap) highlights the importance of structured pathways and standardization (PMID pending—in press).

Registries and collaborative learning networks play a critical role in training. They provide a mechanism for monitoring procedural safety and outcomes while also documenting the progression of operator competence.^117^

In conclusion, the emergence of PFA marks a pivotal moment in AF therapy. Its clinical promise is clear, but its long-term success depends on how it is taught and practiced. Structured training pathways, competency milestones, institutional responsibility, and fellowship integration are indispensable for its safe adoption. By embedding PFA training within the broader framework of electrophysiology education and professional standards, the technology can be adopted responsibly, safeguarding both patient outcomes and the integrity of the field.

## Future directions

### Innovations in pulsed field ablation technology

Continuous efforts are being made to develop and validate new waveforms, as well as to define the adequate PFA dose to obtain effective lesions while preserving safety.^155,156^

#### Focal vs. large footprint innovations and dual-energy ablation

Large footprint PFA platforms (e.g. lattice tip, multielectrode) enable rapid creation of contiguous lesion sets. Dual-energy systems that toggle between pulsed field and RF leverage complementary biophysics to enhance lesion continuity and transmurality while preserving manoeuvrability.^34,45,157^ In this context, lesion stacking (repeat applications along identical trajectories or sequential PF→RF dosing) has shown high acute efficacy with encouraging durability.^34,35,45,157^

#### 3D mapping integration, real-time signal analysis, lesion-effect markers

**Figure 8:**
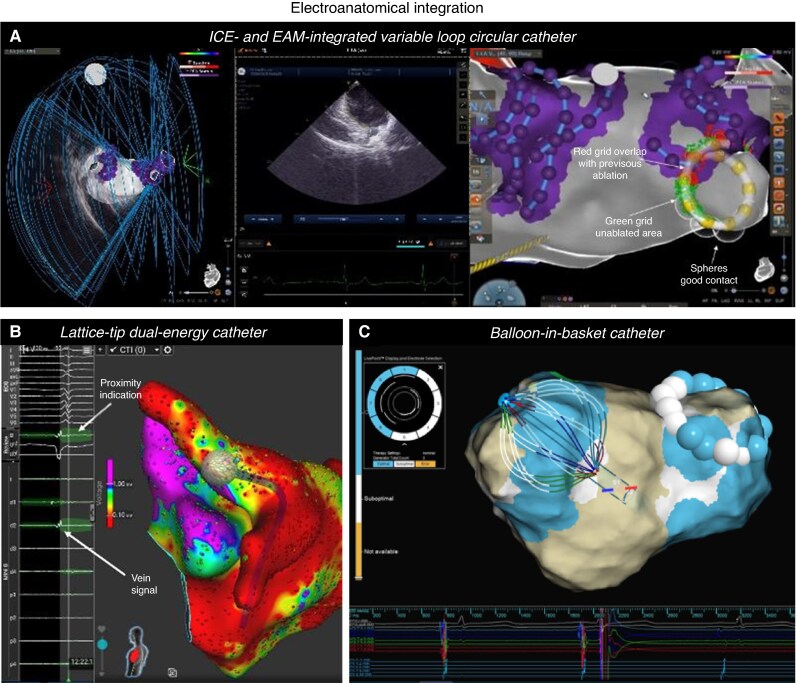
Examples of electroanatomical integration of different pulsed field ablation systems. (*A*) Left and central images: 3D anatomical volume reconstruction using sequential intracardiac echocardiography projections. The image on left side shows ablated zones using the variable-loop circular catheter in purple. Right image: spheres give accurate indication of electrode contact (tissue proximity indicator): sphere dimension is proportional to amount of contact. Small dots predict where energy will be delivered; red indicates lesions overlapping, green unablated tissue. (*B*) Pulmonary vein signals were detected in a redo case using a large footprint lattice-tip catheter. The lattice tip enables high-resolution recording of near-field signals while effectively filtering out noise and far-field activity. Moreover, ablation can be performed directly with the same catheter. The figure also shows the proximity indicator for the single electrode, which helps ensure both accurate signal acquisition and effective lesion formation during ablation. (*C*) Balloon in basket PFA system with electroanatomical mapping integration (LivePoint™ Display Algorithm), which allows for the evaluation of impedance-based lesion-effect parameters through AutoMark lesions (that follow the Livepoint software colour legend) and eField integration. Energy field (eField) displays projection of the electric field of therapy delivery on the map. ICE, intracardiac echocardiography; PFA, pulsed field ablation.

The integration of PFA catheters within three-dimensional EAM systems with sufficient resolution may enable the identification of residual PV connections and confirmation of effective ablation in non-PV regions, while also reducing fluoroscopy exposure.^158,159^ Furthermore, EAM integration may allow for the evaluation of lesion-effect parameters which are associated with transmural and effective ablation lesion formation^160^ (*Figure 8*).

### Ongoing research and clinical trials

Randomized trials such as REPEAT-AF (NCT06199180) will provide important head-to-head comparisons between PFA and thermal ablation in patients with recurrence of AF after initial ablation, a field where data on PFA is currently scarce; however, this represents only a small fraction of a rapidly expanding research landscape, with more than 140 registered PFA studies in patients with AF alone. Beyond AF, early focal PFA series for typical flutter and first clinical experiences in ventricular arrhythmias suggest procedural efficiency with encouraging short-PFA series for typical flutter and first clinical experiences in ventricular arrhythmias suggest procedural efficiency with encouraging short-term outcomes, though standardized endpoints and longer follow-term outcomes, though standardized endpoints and longer follow-up remain priorities.^161,162^

### Emerging applications for pulsed field ablation

#### Posterior wall isolation

Radiofrequency ablation of the PW is limited by concerns of thermal injury to the oesophagus and suboptimal lesion transmurality,^163,164^ limitations that may be addressed by PFA.

**Figure 9:**
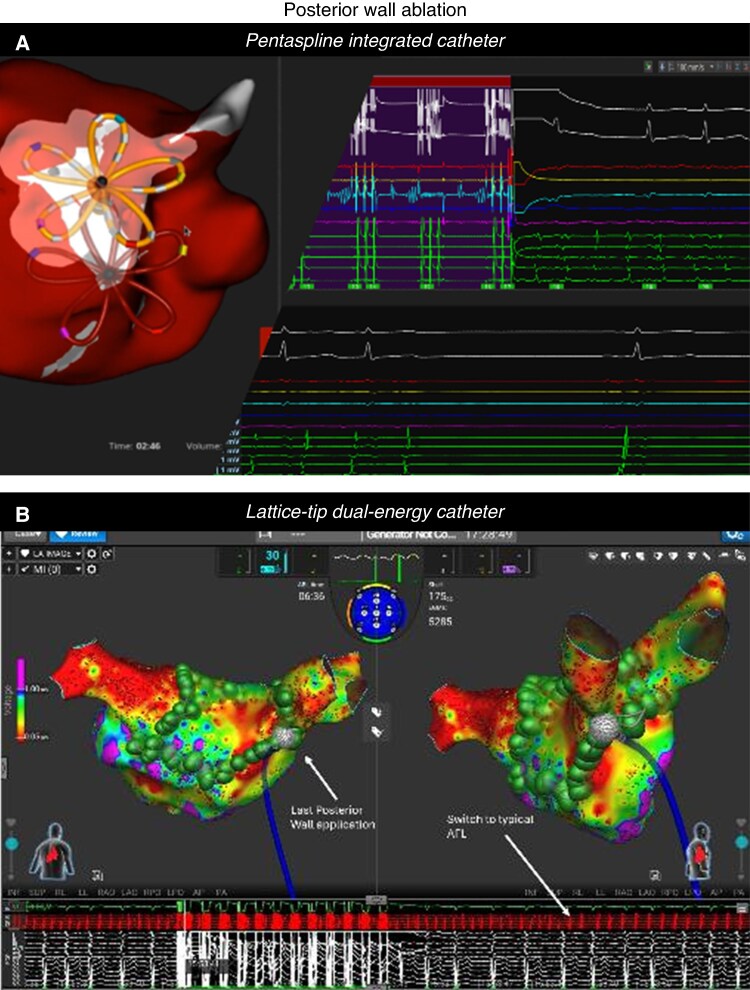
Examples of PW ablation using different electroanatomical integrated PFA systems. (*A*) Example of sinus rhythm restoration after PFA application in the PW. In orange, the integrated pentaspline catheter (Farawave) in the new position, in red the shape of the c FIELDTAG, automatically detecting when and where PFA is delivered and helping to find gaps. (*B*) The image illustrates an example of persistent atrial fibrillation termination during PW ablation, followed by conversion to typical AFl.

In a proof-of-principle study, lesion transmurality following endocardial PW PFA with a pentaspline catheter was verified via direct epicardial mapping.^165^ Several clinical studies have subsequently demonstrated that PW ablation is feasible with several PFA systems, including a pentaspline catheter (*Figure 9*),^43,119,166^ a large-footprint lattice-tip catheter,^45,157^ and a spherical array catheter.^167^ Notably, no evidence of oesophageal injury has been reported across these studies, and procedure times have remained short.

Retrospective, observational data from the MANIFEST-PF registry suggested that adjunctive PW ablation was not associated with improved 12-month freedom from recurrent AF compared to PVI.^168^ Future investigations will be necessary to assess the impact of PW ablation on AF burden, such as the PIFPAF-PFA study (NCT05986526).

#### Superior vena cava isolation

Initial clinical data suggest that superior vena cava isolation can be effectively achieved using either the pentaspline PFA catheter^54^ or a circular multielectrode array PFA catheter.^169^ Future studies are warranted to evaluate the clinical role of both empirical and targeted (when superior vena cava triggers are identified during the procedure) vena cava isolation in terms of rhythm outcomes, particularly given that safety profiles may differ between PFA platforms.

#### Mitral isthmus line

**Figure 10:**
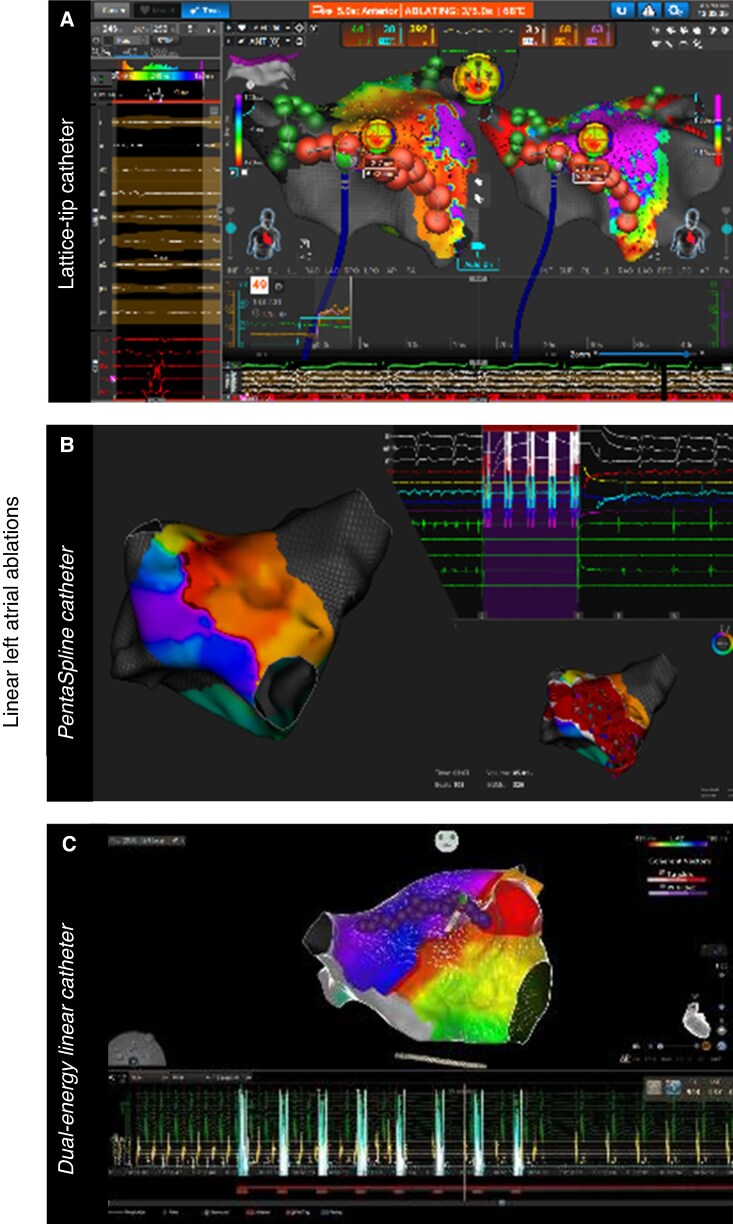
Examples of linear left atrial ablation using different electroanatomical integrated PFA systems. (*A*) The large focal tip catheter also creates broader lesions, thereby facilitating the formation of continuous linear lesions in the left atrium. (*B*) Example of atypical flutter mapped with Farawave 2.0 and Faraview software. On the left, the LAT map that shows a mitral isthmus-dependent atypical flutter; on the right, energy delivered on the anterior mitral isthmus results in flutter termination. (*C*) Successful PFA performed on the critical isthmus located in the anterior roof, as identified by LAT map using the Coherent software module. PF index 500, ITD ≤ 6 mm to ensure contiguity and lesion durability.

Perimitral macroreentrant flutter usually follows prior AF ablation or, less commonly, presents *de novo*.^170^ It is often associated with low-voltage zones, especially at the anterior wall,^171^ which facilitate localized or macroreentrant circuits.^170^ Both anterior and posterior mitral isthmus ablation lines present distinct challenges.^172,173^ Incomplete block predisposes to recurrent perimitral or biatrial flutter.^170–174^ Linear PFA may overcome these limitations by achieving deeper, more uniform lesions with reduced fat or scar attenuation and preserved microvasculature, although periannular coronary spasm remains a concern (*Figure 10*).

Several single-arm studies have evaluated lateral mitral isthmus ablation using the pentaspline catheter (Farapulse, Boston Scientific). Early small studies reported high acute bidirectional block rates, but with 4–10% clinical and up to 40% subclinical coronary artery spasm.^175–177^ A larger study showed that clinical spasm could be prevented with 1–2 mg IV isosorbide dinitrate.^177^ However, in a recent large study, despite 100% acute block, dormant conduction was observed in 14.8% of patients after adenosine,^178^ and remapping at the time of LAA occlusion showed durable block in only 5.5%. Consistently, a prospective registry analysis comparing anterior mitral isthmus line ablation using pentaspline PFA vs. RFA demonstrated greater procedural efficiency with PFA but similarly high rates of line reconnection and comparable mid-term arrhythmia recurrence. These findings suggest that PFA using the pentaspline catheter does not ensure persistent anterior or lateral mitral isthmus block.^179^

The large-footprint dual-energy lattice-tip catheter was evaluated in a dose-finding study combining RF and PFA for lateral mitral isthmus ablation, achieving 100% acute success, with only one adverse event.^34^ Remapping in 69% of patients showed 68% durability. Similar outcomes were reported in the randomized Sphere Per-AF trial with 100% acute success and a 1.4% adverse event rate.^45^ Two recent real-world studies also reported high acute success using this catheter for anterior and lateral mitral lines with short transpired ablation times and low complications rates.^157^ Similar outcomes were confirmed under deep sedation.^180^

Currently, clinical data on acute and long-term outcomes for focal small-tip or other large-tip regional PFA catheters remain limited. Further studies are needed to define optimal catheter design, energy settings, prevent subclinical coronary spasm, and assess long-term durability.

#### Left atrial appendage isolation

Early clinical data suggest that acute isolation of LAA is feasible using the pentaspline PFA catheter, requiring an average of 16 applications in both the flower and basket configurations, with a low risk of complications.^178^ However, systematic remapping at 3 months demonstrated a 95% rate of LAA reconnection, indicating that lesion durability remains a significant limitation.

#### Scar homogenization

A tailored substrate-based approach in patients with AF and advanced atrial substrate including scar homogenization may improve outcomes, as shown in the ERASE-AF trial. A recent single-centre study showed this approach to be safe and effective with a focal PFA system.^181^ Acute procedural success was 100% with 64% freedom from arrhythmias after 6 months. However, larger randomized trials on this matter are still missing.

#### Use in typical atrial flutter

Cavotricuspid isthmus ablation is advised for the management of CTI-dependent AFl, whether clinically documented or encountered during AF ablation.

The pentaspline catheter was initially used off-label for CTI ablation due to ease of use and reported high acute success, although long-term efficacy was not systematically assessed. In a large single-centre experience using predominantly a pentaspline PFA catheter, CTI-dependent flutter ablation achieved high acute success (99.5%) with low short-term recurrence (≈1.5% after blanking).^182^ However, PF-induced right coronary artery spasm—occasionally with malignant ventricular arrhythmias—has been reported.^104,183^ High-dose IV nitroglycerine prevented spasm in most but not all cases.^104,184^ As such, CTI ablation with the pentaspline catheter is advised only with IV nitroglycerine, and its long-term efficacy remains uncertain.^161^

The large-footprint lattice-tip catheter (Sphere-9, Affera) showed 100% acute success in a dose-finding study, mainly using RF energy.^34^ Similar outcomes were confirmed in the randomized Sphere Per-AF trial.^45^ In a real-world study, CTI ablation was performed using RF alone (71%), RF + PF (13%), or PF alone (16%), with 100% acute success and no complications. Although acutely effective and safe, most procedures relied on RF, and further data are needed regarding PF-only safety and coronary spasm risk.

A focal PFA catheter (Farapoint, Farapulse–Boston Scientific) has been evaluated for CTI ablation. Initial studies showed consistent right coronary vasospasm, largely prevented by IV nitroglycerine.^184^ In the ADVANTAGE-AF Phase 2 study,^44^ 141 patients underwent PVI and PW ablation with the pentaspline catheter, followed by CTI ablation using the focal PFA catheter with 4 ± 2 mg IV nitroglycerine. Acute bidirectional block was achieved in almost all patients without complications and flutter recurrence rate was low. These results support the safety and efficacy of focal PFA CTI ablation with nitroglycerine prophylaxis.

Another focal PFA system (CardioFocus, Marlborough, MA) was recently compared to RF in CTI ablation. First-pass block was more frequent and procedure time shorter with PFA, but 5% developed transient ST elevation and 2% transient complete AV block in spite of IV nitroglycerine prophylaxis.^154^

In summary, CTI ablation with PFA currently seems to be limited by proximity-induced right coronary artery spasm and should only be performed under high dose of IV nitroglycerine prophylaxis.

#### Use in ventricular arrhythmias

Catheter ablation of ventricular arrhythmias is often limited by insufficient RF energy penetration in scarred or fatty tissue and the presence of extensive substrate. Preclinical data suggest that PFA lesion formation is not attenuated in these conditions and is rapid, making it particularly promising for ventricular arrhythmia ablation.

Early feasibility studies using focal contact force–sensing catheters with the CENTAURI™ PFA generator (CardioFocus, Marlborough, MA) demonstrated reasonable acute and mid-term success in PVC ablation (initial cohort of 20 patients).^185^ A second 44 patient cohort reproduced the PVC ablation results, but 3-month VT-free survival was only 52% in those with scar-related VT and transient conduction system block occurred in 3 patients (7%), likely due to current leakage.^186^

The large-footprint lattice-tip catheter (Sphere-9™; Affera™) was evaluated in two early small studies for VT ablation using combined RF and PF energy, demonstrating feasibility and safety with acceptable acute and short-term outcomes.^187,188^ In the large multicentre European AVAAR registry, acute and mid-term success rates were reasonable and major peri-procedural complications occurred in only 6%.^189^

Recent prospective data further evaluated focal PFA for PVC and VT ablation in 35 patients. Acute success was high (91%), despite nearly one-third having failed prior RF ablation. PVCs mainly originated from the outflow tracts, and most VTs were ischaemic. Over ∼9 months of follow-up, clinical success was 75% for PVCs but only 45% for VTs, with five patients requiring repeat ablation. Complications included two transient conduction system blocks and two cerebrovascular events (overall major complication rate 6%). These findings suggest good acute efficacy for PVCs but continued uncertainty regarding durability and safety in scar-related VT.^190^

Preclinical data^35^ show that sequential, colocalized RF and PFA significantly increases ventricular lesion depth and width compared with either modality alone, supporting hybrid energy or lesion-stacking strategies for thick or fibrotic myocardium. Consistently, the first-in-human VCAS trial^191^ demonstrated that focal high-voltage PFA can achieve transmural ventricular substrate modification with high acute success and VT burden reduction, although safety events and uncertain durability highlight the need for further controlled studies.

In summary, these findings support the feasibility of the Sphere-9™ ablation system for ventricular arrhythmia ablation, with a reasonable acute and short-term efficacy and safety profile. Larger, controlled studies will be essential to determine lesion durability, procedural safety, and the appropriate clinical role of PFA in ventricular arrhythmias.

#### Use in supraventricular tachycardia

Traditional RF ablation is highly effective and safe for the treatment of paroxysmal supraventricular tachycardia (PSVT). Pulsed field ablation may offer shorter procedure times, provided efficacy and safety are at least equivalent.

To date, focal PF systems have been studied for PSVT ablation in China. In the largest multicentre cohort, initial PFA followed by PF or RF ablation using a focal contact force-sensing catheter (PulsedFA FocalPoint™, JJET) and a 3D cardiac PFA system with magnetic navigation (JJET, China) demonstrated high acute and mid-term success with infrequent reversible AV block.^192^ In a separate study using the same PulsedFA FocalPoint™ catheter, 100% acute and 6-month success with no complications was reported in patients with AVNRT.^193^ A more recent study with a different focal bipolar PFA catheter with contact force-sensing (Pulse Magic™ TrueForce™, MicroPort, China) reported also 100% acute and 6-month success but also transient AV block in 17.5% of patients^14^

In summary, clinical data on PF ablation for PSVT remain limited, and further studies are needed to establish its safety and efficacy.

### Gaps in evidence

Evidence for PFA is most mature for PVI, while substantial uncertainties remain for non-vein isolation, while key uncertainties persist across non-PV targets. Importantly, PFA does not represent a uniform class effect, as substantial heterogeneity exists across technologies with respect to waveform design, voltage, pulse duration, catheter geometry, and application strategies, potentially exceeding the variability observed between RFA approaches. Lesion durability outside the PV, including CTI and left atrial linear lesion, has been characterized mainly in small series and repeat atrial linear lesion has been characterized mainly in small series and redo procedure cohorts. More data from prospective systematic remapping studies with standardized endpoints are needed to better define long-procedure cohorts. Prospective remapping with standardized endpoints is needed to define long-term effectiveness.^47,99^ Safety signals are generally favourable, but rare risks remain insufficiently quantified over extended follow-up, particularly with respect to coronary vasculature, autonomic structures, renal sequelae, and interactions with intracardiac leads or left-up, particularly with respect to coronary vasculature, autonomic structures, renal sequelae, and interactions with intracardiac leads or LAA occluders.^47,99^ Whether specific patient subgroups, such as those with obesity or inflammatory heart disease, derive differential benefit or risk from PFA remains unknown. Moreover, the clinical benefit and durability of PW ablation using PFA have not yet been established. Methodological gaps include dose–response (field strength, pulse number, catheter–tissue coupling), waveform definition and cross-tissue coupling, waveform definition and cross-vendor standardization and inter-vendor standardization, and inter-system comparability given heterogeneous waveforms and catheter geometries.^194^ In contrast to RFA, where surrogates such as contact force, impedance drop, temperature, and stability are established, PFA-specific procedural variables predictive of irreversible lesion formation remain to be defined and validated. Although tissue selectivity underpins the rationale for PFA, quantitative profiles across myocardium, nerve, oesophagus, and vascular tissue require harmonized preclinical and clinical frameworks; biomarkers and imaging markers of damage (e.g. troponin, late gadolinium enhancement on MRI-clinical and clinical frameworks) and biomarkers of damage (e.g. troponin, MRI LGE) and of effect (e.g. durable conduction block metrics) should be anchored to adjudicated outcomes.^194,195^ Additional priorities include mapping integration and validation of linear lesions, including whether criteria derived from RFA translate; optimization of blanking-period criteria; characterization of silent cerebral events and air; effects on coronary perfusion, autonomic tone, and renal function; health-related quality of life relative to thermal ablation; radiation exposure in real-world workflows; cost-effectiveness; and global access.^41,47,99,194^

Health economic data for PFA remain limited.^196^ While potential efficiency gains related to shorter procedures, and lower complication rates may offset higher upfront device costs, robust cost-effectiveness analyses comparing PFA with established thermal modalities across different healthcare systems are lacking. Prospective studies incorporating procedural costs, repeat interventions, resource utilization, and long-term outcomes are required to define the economic value of PFA. While PFA appears easier to use and safer in most hands, current evidence does not yet demonstrate superior effectiveness compared with thermal energy sources. Addressing these questions will require head-to-head platform trials, standardized lesion-head platform trials, standardized lesion assessment protocols, and especially longer follow-up to align efficacy, safety, and value across indications.^41,47,99,161,162,194,195^

## Writing committee position

The writing committee of this joint EHRA scientific statement recognizes PFA as a substantial technological evolution in the interventional treatment of patients with AF. The non-thermal mechanism of PFA, based on cell membrane electroporation, results in efficient myocardial ablation while minimizing collateral damage to critical extracardiac structures. This represents a transformation, a departure from thermal ablation, and a significant advance in the management of patients with AF undergoing catheter ablation.

Based on the available preclinical and clinical evidence, the committee concludes that PFA achieves at least comparable outcomes with a reduced risk of oesophageal injury, PV stenosis, and phrenic nerve damage. Randomized trials and real-world registries consistently demonstrate procedural efficiency, short learning curves, and reproducible lesion durability. However, the committee underscores that the variability in catheter design, waveforms, and dosing variables across platforms warrants cautious, evidence-driven adoption of different systems. The writing committee advocates for the integration of PFA into standard electrophysiology practice within structured, competency-based training programmes. Operators should have a comprehensive understanding of electroporation principles, device-specific workflows, and the management of PFA-related complications such as coronary vasospasm and haemolysis. Institutions implementing PFA should maintain infrastructure that supports procedural monitoring, ensures device compatibility, and enables participation in registries to foster continuous data collection and quality improvement.

The committee highlights that PFA is an adjunct to, not a substitute for, comprehensive electrophysiological expertise within an integrated arrhythmia management platform. Future research should focus on long-term lesion durability, outcomes in patients with persistent AF and patients undergoing repeat procedures, optimization of energy parameters, and the role of PFA in treating non-PV targets and ventricular arrhythmias.

### Summary of key points

- Mechanism of action: PFA represents a technological paradigm shift in AF ablation, utilizing irreversible electroporation to achieve myocardial ablation while preserving adjacent structures such as the oesophagus, phrenic nerve, and vasculature.
- Technology: Multiple PFA platforms are available, and differences in waveform, energy dosing, and catheter design necessitate platform-specific procedural standardization.
- Efficacy: Randomized trials and large registries demonstrate that PFA provides freedom from AF recurrence at least comparable to RF or cryoballoon ablation, with faster procedure times and a short learning curve. Nonetheless, durable lesion formation beyond the PVs, long-term effectiveness, and performance in complex substrates remain incompletely defined.
- Safety: PFA has shown an improved safety profile, with no reported cases of AEF or significant PV stenosis among >500 000 procedures performed globally. However, the true event rate cannot yet be considered zero, and continued vigilance is warranted. Rates of tamponade, stroke, and vascular access complications are low and comparable to thermal ablation. Rare but serious complications, including coronary vasospasm, haemolysis, autonomic effects, and delayed malignant arrhythmias, have been reported, and true event rates require continued surveillance and longer follow-up.
- Clinical indication: PFA is established for *de novo* paroxysmal AF ablation. In persistent AF and repeat procedures, where PFA is also increasingly used, more data including randomized trials is needed.
- Procedural workflow: Both GA and deep sedation are feasible and can be integrated into an efficient and safe PFA workflow. Fluoroscopy-only, 3D mapping, and ICE-integrated workflows are all viable options and dependent on case complexity, and availability.
- Training: Dedicated PFA education should be integrated into electrophysiological curricula, emphasizing the theoretical understanding, procedural competence, and awareness of PFA-related complications.
- Future directions: Priorities include long-term outcome data in different patient strata including health-related quality of life relative to thermal ablation, optimized dosing strategies, standardized training and reporting, cost-effectiveness, sex-specific research, and global access–related quality of life relative to thermal ablation effectiveness.

### Conclusion

In summary, the joint EHRA writing committee supports the responsible clinical adoption of PFA as first-line technology for patients with AF undergoing PVI. Continued global collaboration, multicentre research, transparent data reporting, and harmonized training standards will be essential to ensure the safe, effective, and equitable implementation of this transformative technology. Understanding PFA biophysics and tissue biological responses, its limitations and appreciation of the differences between PFA and thermal energy-based ablations are essential. The writing committee also calls for disclosure of waveforms and technical details by different manufacturers to enable greater and faster progress and maximizing safe implementation of PFA into clinical practice.

## Data Availability

This scientific statement is based on published literature, registry data, and expert consensus. No new patient-level data were generated or analysed for this work. All supporting information is available in the cited references.
